# Food-seeking behavior is triggered by skin ultraviolet exposure in males

**DOI:** 10.1038/s42255-022-00587-9

**Published:** 2022-07-11

**Authors:** Shivang Parikh, Roma Parikh, Keren Michael, Lior Bikovski, Georgina Barnabas, Mariya Mardamshina, Rina Hemi, Paulee Manich, Nir Goldstein, Hagar Malcov-Brog, Tom Ben-Dov, Ohad Glaich, Daphna Liber, Yael Bornstein, Koral Goltseker, Roy Ben-Bezalel, Mor Pavlovsky, Tamar Golan, Liron Spitzer, Hagit Matz, Pinchas Gonen, Ruth Percik, Lior Leibou, Tomer Perluk, Gil Ast, Jacob Frand, Ronen Brenner, Tamar Ziv, Mehdi Khaled, Shamgar Ben-Eliyahu, Segev Barak, Orit Karnieli-Miller, Eran Levin, Yftach Gepner, Ram Weiss, Paul Pfluger, Aron Weller, Carmit Levy

**Affiliations:** 1 Department of Human Genetics and Biochemistry, Sackler Faculty of Medicine, Tel Aviv University, Tel Aviv, Israel; 2 Department of Human Services, The Max Stern Yezreel Valley Academic College, Yezreel Valley, Israel; 3 The Myers Neuro-Behavioral Core Facility, Tel Aviv University, Tel Aviv, Israel; 4 School of Behavioral Sciences, Netanya Academic College, Netanya, Israel; 5 Endocrine Service Unit, Sheba Medical Center Hospital, Tel Hashomer, Ramat Gan, Israel; 6 School of Public Health, Sackler Faculty of Medicine and Sylvan Adams Sports Institute, Tel Aviv University, Tel Aviv, Israel; 7 Department of Otolaryngology, Head and Neck surgery, Meir Medical Center, Kfar Saba, Israel; 8 Zuckerman Mind Brain Behavior Institute, Howard Hughes Medical Institute and Department of Biochemistry and Molecular Biophysics, Columbia University, New York, NY USA; 9 School of Zoology, Faculty of Life Science, Tel Aviv University, Tel Aviv, Israel; 10 Division of Dermatology, Tel Aviv Sourasky (Ichilov) Medical Center, Tel Aviv, Israel; 11 Phototherapy Unit, Assuta Medical Center, Tel Aviv, Israel; 12 Sackler School of Medicine, Tel Aviv University, Tel Aviv, Israel; 13 Division of Endocrinology, Chaim Sheba Medical Center, Tel Hashomer, Israel; 14 Department of Plastic and Reconstructive Surgery, E. Wolfson Medical Center, Holon, Israel; 15 Institute of Oncology, E. Wolfson Medical Center, Holon, Israel; 16 The Smoler Proteomics Center, Lorry I. Lokey Interdisciplinary Center for Life Sciences and Engineering, Technion, Haifa, Israel; 17 INSERM 1279, Gustave Roussy, Université Paris-Saclay, Villejuif, France; 18 School of Psychological Sciences, Tel Aviv University, Tel Aviv, Israel; 19 Sagol School of Neuroscience, Tel Aviv University, Tel Aviv, Israel; 20 Department of Medical Education, Sackler Faculty of Medicine, Tel Aviv University, Tel Aviv, Israel; 21 Department of Pediatrics, Ruth Rappaport Children’s Hospital, Rambam Medical Center and Technion School of Medicine, Haifa, Israel; 22 Research Unit Neurobiology of Diabetes, Institute for Diabetes and Obesity, Helmholtz Zentrum München, German Centre for Diabetes Research (DZD), Neuherberg, Germany; 23 Department of Psychology and the Gonda Brain Research Center, Bar-Ilan University, Ramat Gan, Israel

**Keywords:** Cell biology, Neuroendocrinology, Feeding behaviour

## Abstract

Sexual dimorphisms are responsible for profound metabolic differences in health and behavior. Whether males and females react differently to environmental cues, such as solar ultraviolet (UV) exposure, is unknown. Here we show that solar exposure induces food-seeking behavior, food intake, and food-seeking behavior and food intake in men, but not in women, through epidemiological evidence of approximately 3,000 individuals throughout the year. In mice, UVB exposure leads to increased food-seeking behavior, food intake and weight gain, with a sexual dimorphism towards males. In both mice and human males, increased appetite is correlated with elevated levels of circulating ghrelin. Specifically, UVB irradiation leads to p53 transcriptional activation of ghrelin in skin adipocytes, while a conditional p53-knockout in mice abolishes UVB-induced ghrelin expression and food-seeking behavior. In females, estrogen interferes with the p53–chromatin interaction on the ghrelin promoter, thus blocking ghrelin and food-seeking behavior in response to UVB exposure. These results identify the skin as a major mediator of energy homeostasis and may lead to therapeutic opportunities for sex-based treatments of endocrine-related diseases.

## Abstract (web summary)

Ultraviolet exposure on the skin promotes food intake and body weight gain in males, but not females, by increasing ghrelin expression in skin adipocytes.

## Main

Sex differences have profound effects on health and behavior^1^. Yet, whether men and women react differently to environmental cues, such as ultraviolet (UV) radiation, remains under investigated. UV was recognized as a carcinogen in 1928 (ref. ^2^), sparking a massive cultural trend of minimizing exposure to the sun from the mid-1900s^2^. But subsequent epidemiological studies have painted a more complex picture of UV’s role in human health, by indicating that it can extend life expectancy, due to protection against cardiovascular disease and other causes of mortality^2^. Sun exposure increase liver metabolism, protecting the organ from hepatocellular lipotoxicity^3^ and metabolic disease^3^.

The solar radiation’s health benefits have been attributed to vitamin D^4^. But two recent large-scale clinical trials showed that vitamin D alone was not associated with reduced risk of cardiovascular disease, all-cause mortality and invasive cancer^4^. These findings indicate that at least some of the health benefits of sunlight are independent of vitamin D.

The skin is the largest organ in the human body and is the first line of defense against environmental threats. Its epidermal and dermal layers are separated by a basal epidermal layer composed of undifferentiated, self-renewing keratinocytes and melanocytes. The dermis includes fibroblasts and blood vessels, with the hypodermis mainly consisting of adipocytes^5^. The skin is a dermato-endocrine organ, with its resident cells, featuring multiple hormone receptors^6^. With the exception of β-endorphin^7^, vitamin D and estrogen, production and release of hormones from skin cells into the blood has not been documented, nor are the triggers for such activities (reviewed elsewhere^6^) or their potential systemic effect on body physiology known.

Appetite regulation is a profoundly complex process that directly influences health, involves ghrelin and leptin^8^ hormones. Ghrelin modulates the responses to changes in energy homeostasis via binding to growth hormone secretagogue receptor (GHS-R)expressing neurons within hypothalamic nuclei that regulate food intake, body weight, and plasma glucose^8^. The binding of Ghrelin to GHS-R within the hypothalamus activates agouti-related neuropeptide (AgRP)-expressing neurons in the arcuate nucleus to activating the orexogenic pathway via neuropeptide Y (NPY) receptors and to inhibit the anorexogenic pathway activity of neurons that express pro-opiomelanocortin (POMC) and stimulate appetite^9^. Circulating ghrelin levels are at their nadir after a meal, and are increased thereafter^8^. Ghrelin is the only peripheral peptide known to stimulate appetite, but there are many peripheral hormones that suppress appetite including cholecystokinin, Peptide YY, pancreatic polypeptide, insulin, leptin, glucagon-like peptide 1, gastric inhibitory polypeptide, adiponectin and oxyntomodulin^9,10^. Leptin secretion from adipose tissue counters ghrelin’s stimulus by activating POMC-expressing neurons in the hypothalamus, prompting a feeling of satiation^11^. The interplay of AgRP-expressing and POMC-expressing neurons in hypothalamic nuclei involved in energy homeostasis as part of the melanocortin system regulates satiety status, food-seeking behavior and metabolic rate^3^. Ghrelin secretion into the blood is regulated by nutrients and metabolites such as glucose, insulin and long-chain fatty acids, which all inhibit ghrelin secretion and by monosodium glutamate, dopamine, oxytocin and adrenaline, which enhance ghrelin secretion^12^. NF-κB, Nkx-2.2 (ref. ^13^), KLF4 (ref. ^14^) and PAX4 (ref. ^15^) have been implicated in the transcriptional regulation of *ghrelin* expression. Importantly, ghrelin is postulated to regulate hedonic feeding behaviors by increasing the reward value of highly palatable food in rodent models^12^.

The environmental factors that control ghrelin secretion are music^16^, light^17^ and odor^18^, but the underlying mechanisms have yet to be revealed. Although the stomach is the major source of ghrelin secretion^8^, gastrectomy reduces it only by 65%^19^, suggesting that other tissues produce ghrelin. One of these tissues maybe the skin, as ghrelin was found to be expressed in the epidermis and dermis^20,21^, as well as in the hypodermis in subcutaneous adipose tissue^22^.

Here, by analyzing human dietary data of approximately 3,000 people along the year, we reveal that men are significantly affected by solar radiation and its seasonal fluctuation compared to women, resulting in a more pronounced energy intake during summer. Further, we found that daily low-levels of UVB exposure enhance the food intake and food-seeking behavior of male mice, but not of female mice, a sex difference we observed also in human patients undergoing phototherapy. Appetite enhancement was correlated with elevated levels of circulating ghrelin, in both mouse and human males. We further discovered that skin adipocytes produce and release ghrelin after UVB exposure, triggered by the DNA damage-induced activity of p53. Estrogen inhibited p53 activity in adipocytes, thus blocking elevation of ghrelin levels in females. Mice lacking p53 in their adipocytes failed to increase their ghrelin levels in response to UVB and their food-seeking behavior. These data demonstrate that response to UVB radiation is sex dependent. We further elucidate the skin-adipocyte-mediated mechanism underlying this behavioral difference.

## Results

### Solar exposure enhances the energy intake and metabolic profile of men compared to women

**Fig. 1:**
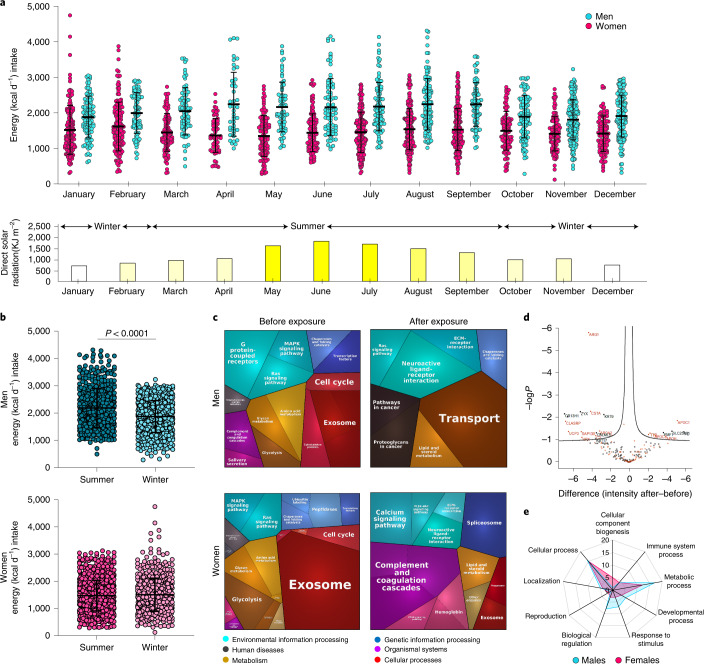
Solar exposure enhances the energy intake and metabolic profile of men compared to women. **a**, Dot plot of the monthly energy intake (Kcal per day), from 1999 to 2001, of 2,991 men (cyan blue) and women (pink) (top). Midline represents the median. Data are presented as mean ± SD. Men’s energy consumption was significantly higher during the summer (2,188 Kcal versus 1,875 Kcal, *p* < 0.001), while energy consumption in women remained constant (1,507 Kcal versus 1,475 Kcal, *p* = 0.795). Lower panel: Monthly average of direct solar radiation (KJ/m); yellow intensity reflects the radiation strength. **b**, Energy intake (Kcal per day) of men (top) and women (lower panel) in winter (October to February) and summer (March to September). Each individual participant is represented by a dot (summer: *n* = 556 men, *n* = 1,045 women; winter: *n* = 774 men, *n* = 616 women). Data are presented as mean ± SD. For the statistical analysis, unpaired *t*-test assuming unequal variance with Welch’s correction was performed. We found that men consume more calories during the summer than in the winter (*p* < 0.001), while the calorie intake of women was similar (*p* = 0.27) between the two seasons, demonstrating that only men are affected by the seasonal change. **c**, Proteomics analysis, shown as Proteomap, illustrates the functional categories of men (top) and women (bottom) blood plasma proteins before (left panel) and after (right) exposure to 2,000 mJ/cm^2^ solar UVB. Data presented in each polygon represents proteins in a single KEGG pathway with >2 fold change (*n* = 5 biologically independent human subjects per condition). **d**, Volcano plot of differentially expressed proteins in men (before/after solar UVB exposure) by log_2_ fold change; metabolic-related proteins are marked orange. **e**, Radar map of Gene Ontology enrichment of differentially expressed proteins identified by mass spectrometry analysis of blood plasma proteins from mice after UVB (50 mJ/cm^2^) or mock UVB (control) irradiation (*n* = 3 biologically independent mice per condition). Source data

**Extended Data Fig. 1:**
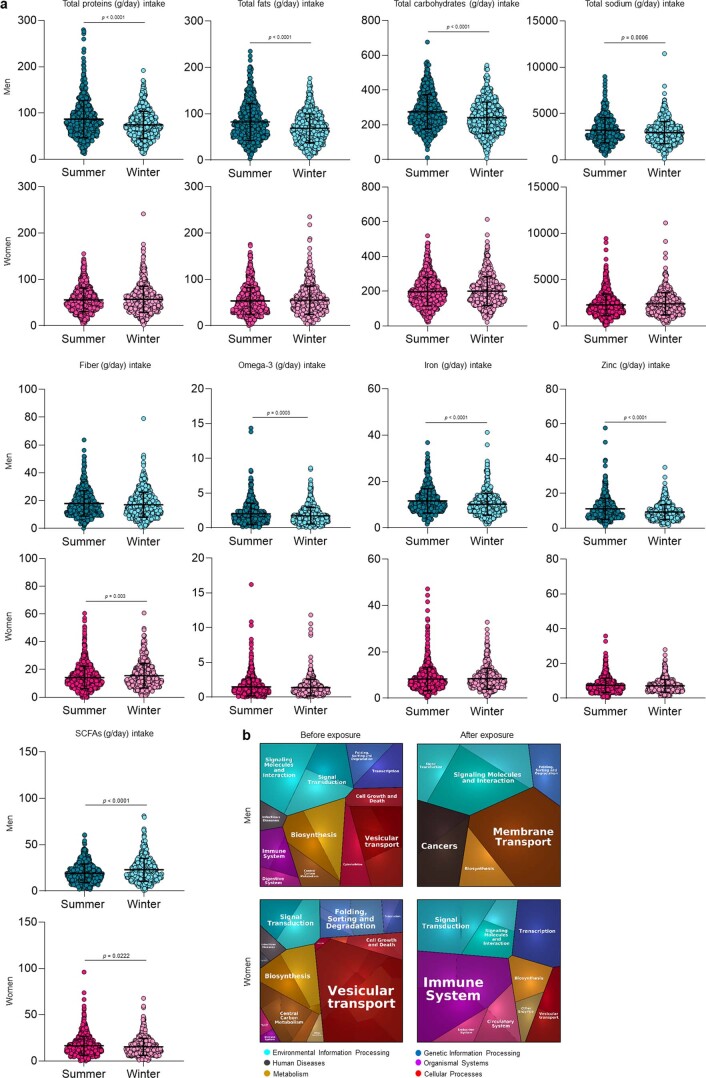
Solar exposure enhances the energy intake and metabolic profile of men compared to women, Related to Fig. 1. **a**, Total protein, fat, carbohydrate, sodium, fiber, omega-3, iron, zinc, and SCFAs intake (in g/day) by men and women in winter (October-February) and summer (March-September). Each participant is represented by a dot (summer: *n* = 556 men, *n* = 1,045 women; winter: *n* = 774 men, *n* = 616 women). Data are presented as mean ± SD. For the statistical analysis, unpaired two-tailed *t*-test assuming unequal variance with Welch’s correction *p*-value for carbohydrates (men *p* <0.0001; women *p* = 0.7069), protein (men *p* <0.0001; women *p* = 0.9076), fat (men *p* <0.0001; women *p* = 0.6185, sodium (men *p* <0.0001; women *p* = 0.0703), fiber (men *p* = 0.076; women *p* = 0.03), omega-3 (men *p* = 0.0003; women *p* = 0.565), iron (men *p* <0.0001; women *p* = 0.7485), zinc (men *p* <0.0001; women *p* = 0.3606), and SCFAs (men *p* <0.0001; women *p* = 0.022) was performed are shown, demonstrating that only men are affected by the seasonal change. **b**, Proteomics analysis, shown as Proteomap, illustrating the detailed hierarchy of functional categories (signal transduction, immune system, vesicular transport, digestive system, circulatory system, biosynthesis, folding, sorting and degradation) of men (upper panel) and women (lower panel) blood plasma proteins before (left panel) and after (right panel) exposure to 2,000 mJ/cm^2^ solar UVB. Data presented in each polygon represents proteins in a single KEGG pathway with >2-fold change (*n* = 5 biologically independent human subjects per condition). Source data

A meta-analysis of several research studies has found differing results for the influence of seasonality on food intake^23^. We, therefore, analyzed data from a 3-year national nutrition survey of approximately 3,000 people. Using a generalized linear model adjusted for age, we found a significant interaction (*P* <0.001) between sex and season, revealing that men are markedly affected by solar radiation and its seasonal fluctuation compared to women (Fig. 1a). Additionally, we averaged the monthly direct solar radiation data (KJ/m^2^) (Fig. 1a) and found that men significantly increase energy consumption during the summer (March to September) as compared to winter (October to February) (2,188 Kcal versus 1,875 Kcal, respectively; *p* <0.001), while energy consumption in women remains the same (1,475 Kcal versus 1,507 Kcal, respectively; *p* = 0.79) (Fig. 1b and Extended Data Fig. 1a). Notably, since we found a significant increase in nutrients including: carbohydrates, proteins, fat, sodium, omega-3, zinc and iron in men during the summer (Extended Data Fig. 1a), it is possible that the increase in men’s energy intake is due to an increase in appetite-stimulating nutrients such as sodium.

**Extended Data Fig. 2:**
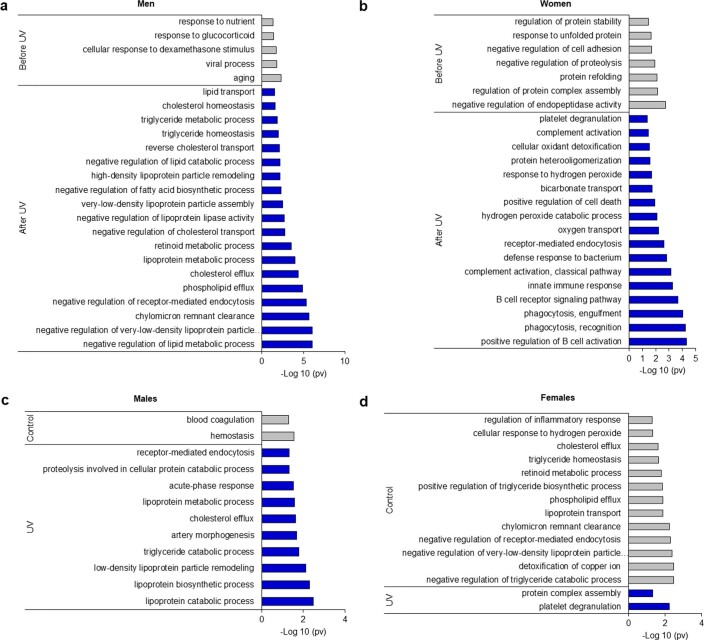
Solar exposure enhances the energy intake and metabolic profile of men compared to women, Related to Fig. 1. **a**,**b**, Horizontal bar graphs represent significantly (*p* <0.05) enriched GO biological processes identified using the Gene Ontology tool for differentially expressed blood plasma proteins of (**a**) men and (**b**) women following a single solar exposure (UVB 2,000 mJ/cm^2^) (*n* = 5 biologically independent human subjects per condition). **c**,**d**, Horizontal bar graphs represent significantly (*p* <0.05) enriched GO biological processes identified using the Gene Ontology tool for differential expressed blood plasma proteins of (**c**) male and (**d**) female mice after 10 weeks of daily UVB exposure (50 mJ/cm^2^) (*n* = 3 biologically independent mice per condition). Source data

To further explore the difference between the response of men and women to solar exposure, we asked volunteers (*n* = 5 men and *n* = 5 women; age 18–55 years) to expose, for about 25 min, to the sun on a bright sunny midday (2,000 mJ/cm^2^ UVB). Plasma samples taken before and after the exposure were subjected to mass spectrometry (Supplementary Table 1), followed by functional relevance by proteomap and Gene Ontology (GO) analysis. Upon solar exposure, in men there is an enhancement of lipid and steroid metabolism while in women a decrease is observed (Fig. 1c and Extended Data Figs. 1b and 2a,b). Further, metabolism-associated peptides were the most significantly affected by the solar exposure, including enrichment in key Arginase 1 (ARG1) signaling elements of the urea cycle: apolipoprotein C-I (APOC1) signaling, a central regulator of high-density lipoprotein (HDL) metabolism, peroxisome proliferator-activated receptor (PPAR) signaling and cholesterol metabolism (Fig. 1d). Our data demonstrate that, while both sexes increase their response to environmental cues and decrease their extracellular vesicle pathways, in terms of the immune system and metabolism, men and women react in the distinct manner.

**Fig. 2:**
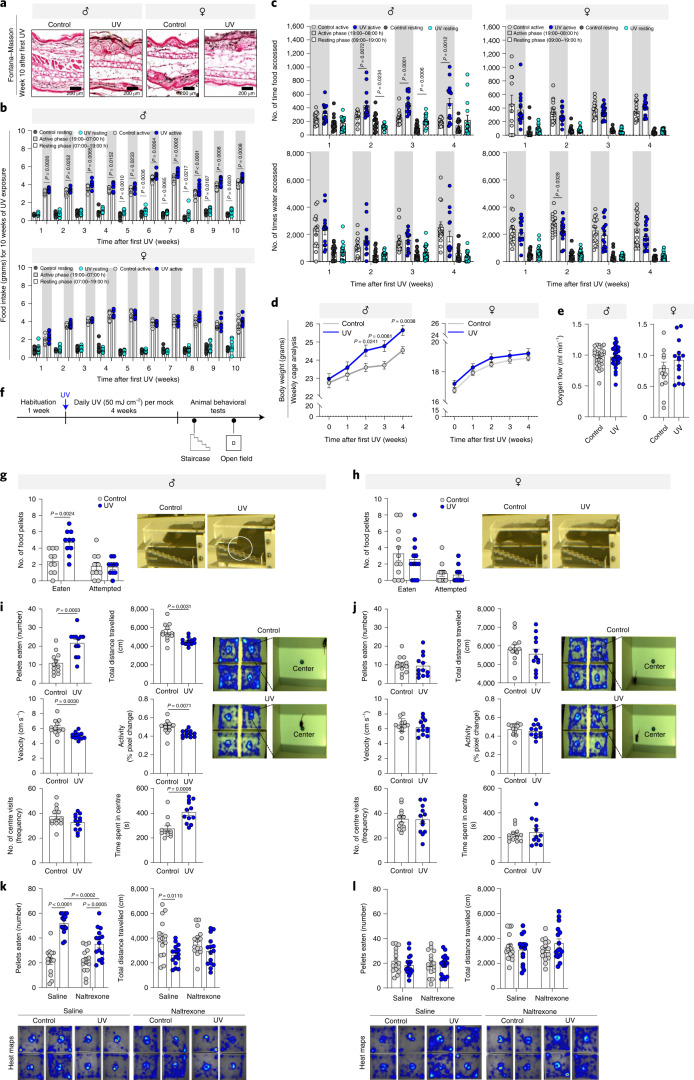
Daily UVB radiation enhances food-seeking behavior in males. **a**, Fontana–Masson staining of representative ear sections of mice exposed to daily UVB (50 mJ/cm^2^) or mock-UVB mice (control). **b**, Weekly food intake of standard chow food (in grams) during the resting and active phases. (*n* = 10 biologically independent mice per condition). **c**, PhenoTyper analysis upon daily UVB (50 mJ/cm^2^) or mock-UVB (control) for 4 weeks: number of times accessing food (upper panel) or water (lower panel) during the resting and active phases (*n* = 15 biologically independent male mice per condition; *n* = 16 biologically independent female mice per condition). **d**, Weekly mean body weight (grams) after daily UVB (50 mJ/cm^2^) or mock-UVB (control) irradiation (*n* = 12 biologically independent mice per condition). **e**, Respirometry analysis of UVB (50 mJ/cm^2^) or mock-UVB (control) treated mice 10 weeks after the first UVB treatment. Dot plot represents oxygen flow (ml/min) VO_2_ consumed at rest (*n* = 29 for control and *n* = 28 biologically independent male mice and *n* = 14 biologically independent female mice per condition). **f**, Experimental design for **g**–**j**. **g**,**h**, Staircase test (**g**) male and (**h**) female mice (*n* = 10 biologically independent male per condition and *n* = 12 biologically independent female mice per condition) (left). Representative images from the video of the test session (right). **i**,**j**, Open-field for (**i**) male and (**j**) female mice (*n* = 12 biologically independent mice per condition) (left). Heat maps representative images (right). Far-right: Representative images from the video of the test session. **k**,**l**, Mice irradiated daily with UVB (50 mJ/cm^2^) or mock-UVB (control) for 8 weeks and injected with opioid antagonist (naltrexone (5 mg/kg)) or saline 30 min before the open-field test. Upper panel: Number of sucrose pellets eaten and the total distance travelled by (**k**) male and (**l**) female (*n* = 15 biologically independent male mice per condition and *n* = 16 biologically independent female mice per condition). Representative heat maps (bottom). In all relevant panels, data are presented as mean ± SEM; Two-tailed unpaired *t*-test *p-*values are shown or statistical details for sex or UVB or naltrexone factors in the ANOVAs (*F*-values, degrees of freedom, *p*-value) with interaction appears in Supplementary Table 10 or two-way ANOVA analysis with multiple correction test appears in Supplementary Table 11. Source data

**Extended Data Fig. 3:**
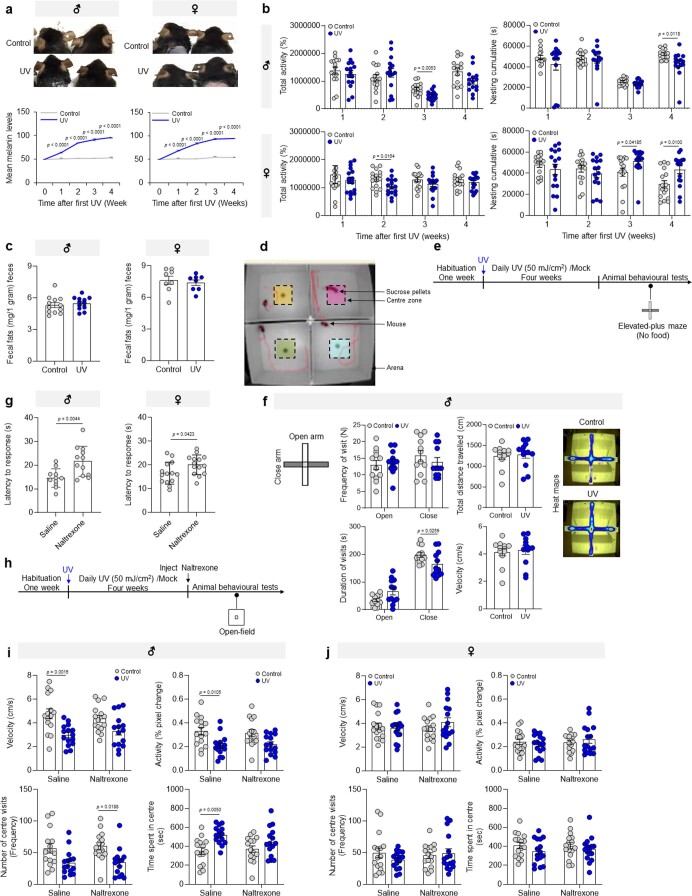
Daily UVB radiation enhances food-seeking behavior in males, Related to Fig. 2. **a**, Upper panel: Photographs of representative mice exposed to daily UVB (50 mJ/cm^2^) or mock-UVB (control). Lower panel: Mean melanin intensity (*n* = 7 biologically independent mice per condition). **b**, PhenoTyper analysis of mice treated daily with UVB (50 mJ/cm^2^) or mock-UVB (control) for 4 weeks. Data represent the total activity within the arena (left panel) and the cumulative nesting duration of time spent (23 h) (*n* = 15 male and n = 16 biologically independent female mice per condition). **c**, Fecal fat analysis of mice 10 weeks after the first daily UVB (50 mJ/cm^2^) or mock-UVB (control) treatments (*n* = 12 male and *n* = 8 biologically independent female mice per condition). Dot plot presents the mean of fecal lipids (in mg per gram of feces). **d**, Representative image from the open-field test (four arenas) trial session video. **e**, Experimental design. **f**, Left panel: Elevated-plus maze for male UVB-treated and control mice (*n* = 12 biologically independent mice per condition). Right panel: Representative heat maps for each condition. **g**, Hot plate test response of male (left panel) and female (right panel) C57BL/6 J mice 30 min after being injected with opioid antagonist naltrexone (5 mg/kg of the bodyweight) or saline (Biologically independent males: *n* = 11 in saline and *n* = 12 mice per condition; Biologically independent females: *n* = 14 mice per condition). **h**–**j**. Habituated mice were irradiated daily with either UVB or mock-UVB (control) and were injected with opioid antagonist (naltrexone (5 mg/kg of the body weight)) or saline 30 min before the open-field test. (**h**) Experimental design. (**i,j**) Left: The velocity, activity and frequency of and the total time spent in the center zone by (**i**) male and (**j**) female (*n* = 15 biologically independent males and *n* = 16 biologically independent female mice per condition). In all relevant panels: Data are presented as mean ± SEM; Two-tailed unpaired t-test *p*-values are shown, or statistical details for sex or UVB or treatment factors in the ANOVAs (*F*-values, degrees of freedom, *p-value*) with interaction appears in Supplementary Table 10, or two-way ANOVA analysis with multiple correction test appears in Supplementary Table 11. Source data

To further investigate the sex-based effect of UVB exposure, we exposed mice (12 mice in each sex) for 10 weeks to daily UVB radiation (50 mJ/cm^2^) approximately equal to 20–30 min of ambient midday sun exposure for a fair-skinned person (Fitzpatrick skin phototypes II–III) in Florida during the summer. A significant ear pigmentation was observed in both males and females following the daily UVB treatment (Fig. 2a and Extended Data Fig. 3a), indicating that the UVB response was persistent. Following the exposure, the total plasma proteins were subjected to proteomic analysis^24^ (Supplementary Table 2). As in humans, following the UVB exposure, in mice males show more protein changes related to metabolism compared to females (Fig. 1e and Extended Data Fig. 2c,d). Taken together, our data indicate that men are more responsive to solar UV and seasonal changes and that the metabolism is significantly affected in men and male mice.

### Daily UVB radiation enhances food-seeking behavior in males

To explore the effect of UVB exposure on metabolic processes in a sex-dependent manner, each mouse was treated with UVB exposure (50 mJ/cm^2^) for 10 weeks. We measured food intake for 10 weeks during the active (19:00–07:00 hours) and resting (07:00–19:00 hours) phases, keeping one animal per home cage. We observed a significant increase in food intake by males upon UVB exposure throughout the dark phase of the 10-week duration of the study (Fig. 2b) and a significant increase during the light phase 5 weeks after the UVB exposure (Fig. 2b). In contrast, female mice showed no significant increase in food intake following UVB exposure (Fig. 2b). Further, we performed PhenoTyper analysis, daily through the 4 weeks of treatment. Dorsally shaved mice were exposed to UVB (50 mJ/cm^2^) or mock treated for 4 weeks. Male mice treated with UVB made significantly more visits to the food zone than controls, whereas no significant difference was observed in the females (Fig. 2c and Extended Data Fig. 3b). This demonstrate that UVB enhances eating behavior in male mice leaving female mice unaffected. Further, the wild-type colony show that chronic UVB exposure resulted in a significant increase in body weight in male mice and not in females (Fig. 2d).

To assess the effect of UVB on metabolic process, we measured the maximum amount of oxygen consumed at physiological level (VO_2_). Dorsally shaved mice were exposed to UVB (50 mJ/cm^2^) for 10 weeks and a control group was mock-UVB treated. There was no significant difference in VO_2_ of either male or female mice following UVB radiation (Fig. 2e), suggesting that basal metabolic rate is not changed by UVB. Further, we found that UVB exposure doesn’t modulate fecal fat content in male and female mice (Extended Data Fig. 3c). Taken together, our data demonstrate that UVB influences food intake, although not metabolism, in males.

Next, we investigated whether the increase in food intake observed in UVB-exposed male mice was reflected in changes in food-related behavior (Fig. 2f). To test the craving, in general and food-seeking behavior, in particular, behavioral models make food difficult to access in order to measure the effort an animal will expend to obtain food^25^. We implemented the classic staircase test, previously used to examine motor deficits in rodents^26^ as calibrated to motivate mice with food^26^. In this test, animals must use reach and grasp movements to pick up and retrieve food pellets^26^.The test sessions revealed that UVB-treated males ate significantly more food pellets than males not exposed to UVB (Fig. 2g). In contrast, UVB-exposed females exhibited a mild decrease in food intake (Fig. 2h). Moreover, the ‘attempt score’, which measures success in reaching out for food pellets, did not change upon UVB treatment for either sex, indicating that UVB exposure does not impact motor functions.

The open-field test is commonly used to monitor anxiety-like behavior and locomotion in mice^27^, while is also suitable for examination of food-seeking behavior in a ‘risky’ environment^25^. In the version of the test we used, the mouse must cross an open space in bright light to obtain food pellets located in the center of the arena (Extended Data Fig. 3d). In comparison to mock-UVB-treated males, UVB-treated males showed a significant increase in the number of food pellets eaten and the time spent in the center as well as a notable decrease in the total distance travelled in the arena, velocity and activity levels during the test session (Fig. 2i). UVB-treated females showed no significant changes compared to their mock-UVB-treated counterparts (Fig. 2j). Further, using PhenoTyper cages which tracks animals for 23 h weekly, we found no significant differences in the total activity or nesting behavior of either males or females when mock- and UVB-treated animals were compared (Extended Data Fig. 3b). These data rules out the possibility that UVB induced behavioral changes related to physical activity.

Since UVB exposure induces β-endorphin production and reduces anxiety-like behavior^7^, we used the elevated-plus maze test to determine whether the effect of daily UVB exposure on male food craving is due to changes in anxiety-like behavior. Exposure to UVB light caused male mice to significantly reduce the frequency of visits and time spent in the closed arms of the maze (Extended Data Fig. 3e,f), but there were no significant differences in the total distance travelled or the velocity of travel through the maze between the UVB-treated and mock-UVB-treated male mice (Extended Data Fig. 3f). These data suggest that, indeed, UVB exposure, in our experimental model, decreases anxiety-like behavior. Although anxiety can affect food intake^28^, the total time spent by males in the center of the arena during the open-field test was three times longer in the presence of food (Fig. 2i) suggesting that, although UVB-treated males may experience less anxiety, the visit to the center is motivated by the food. Further, we repeated the open-field test in mice treated with the β-endorphin antagonist naltrexone (5 mg kg^−1^)^7^ before the test (Extended Data Fig. 3h). Male and female mice exhibited significant increased tolerance to pain after naltrexone treatment (Extended Data Fig. 3g), thus confirming the efficacy of the drug. Both the saline and the naltrexone UVB-treated (50 mJ/cm^2^) males demonstrated an increase in food consumption (Fig. 2k), velocity (Extended Data Fig. 3i), activity levels (Extended Data Fig. 3i) and number of visits to the center of the arena (Extended Data Fig. 3i) than mock-UVB-treated mice. Only saline-injected UVB-treated males and not naltrexone-treated males showed a significant decrease in the total distance travelled in the arena (Fig. 2k) and an increase in the total time spent in the center (Extended Data Fig. 3i) compared to mock-UVB-treated mice. The groups of saline- and naltrexone-injected UVB-treated females did not differ from each other or from mock-UVB-treated females in the measured open-field test parameters (Fig. 2l and Extended Data Fig. 3j). Collectively, our data demonstrate that the male mice’s enhanced food-seeking behavior upon UVB exposure was not altered by the absence of β-endorphin’s agonistic influence on mu-opioid receptors.

### UVB exposure enhances appetite in human males

Does UVB light similarly affect the appetite of humans? To address this question, a cohort of subjects undergoing phototherapy were asked to respond to the Disease-Related Appetite Questionnaire (DRAQ)^29^, before (time point T1) and one month after the first UVB treatment (time point T2). During this time frame, patients were exposed to UVB light (0.1–2.5 J/cm) two or three times a week, for a total of 10–12 UVB exposures. Since skin tone directly affects the amount of UVB that penetrates the skin^30^ and, therefore, probably influences the response, most of the patients in our study had II–III skin tone according to Fitzpatrick Skin Type to avoid this bias. As we focused only on changes in appetite and hunger, we omitted irrelevant items from the DRAQ (for example, those related to food taste), retaining items such as ‘My appetite varies from day to day’ and ‘I feel hungry’. Z-scores results indicated that, unlike females, male participants reported more daily variation in their appetite and a greater frequency of feeling hungry at T2 than at T1 (Table 1). Male patients also reported a lower frequency of eating at T2 compared to T1, though the amount of food consumed during each meal might have been bigger. Female participants showed no significant differences between T1 and T2. Greater variation in appetite, greater frequency of feeling hungry and greater frequency of eating (observed in the humans) is not the same as enhanced appetite (in the mice), however, this human questionnaire data agrees with our animal behavioral data, indicating that following UVB exposure only males experience changes in appetite.

**Table 1:** UVB exposure enhances appetite in human males

	T1		T2		Ties	W	Z	T1		T2		Ties	W	Z
	Median	Range	Median	Range				Median	Range	Median	Range			
Level of appetite	2	3–2	2	3–2	13	0	−1.00^a^	2	3–2	2	3–1	15	2	−0.58^a^
Variation in day to day appetite	3	3–1	2	3–1	8	3.5	−1.63^a^* *P* = 0.051	2	3–1	2	3–1	11	13.5	−0.09^a^
Feeling full only after a full meal	3	3–2	3	3–2	10	5	0.00^b^	2.5	3–1	2	3–1	10	27	1.41^c^
Feel hungry frequently	2	2–1	2	2–1	11	6	−1.73^c^* *P* = 0.0415	2	3–1	2	3–1	15	4.5	−0.82^c^
Eating frequency	2	3–1	2	3–1	11	0	−1.73^a^* *P* = 0.0415	2	3–1	2	3–1	15	4	−0.58^c^
Variation in daily eating	2	3–1	2	3–1	4	25	−0.28^a^	2	3–1	2	3–1	12	9	−0.33^a^

Patients responded to selected DRAQ items at T1, before the first UVB treatment and at T2, after 1 month of UVB treatment (0.1–2.5 J/cm, total of 10–12 UVB exposures during this time frame). The questionnaire included questions concerning appetite, hunger and other psychological eating-related issues. The questions were rated on a three-point multiple-choice scale. Reported are within-group differences (ranks of T1 versus T2 for each sex separately); Wilcoxon tests (*n* = 14 biologically independent males; *n* = 18 biologically independent females).

Range, minimum – maximum; Ties, number of cases where T1 = T2.

W, test statistics of the sums of positive ranks (comparing the sums of the positive and negative ranks).

Z, standard normal distributed z value (to test significance).

^a^Based on positive ranks.

^b^The sum of negative ranks equals the sum of positive ranks.

^c^Based on negative ranks.

### UVB radiation induces ghrelin production and secretion in skin adipocytes

**Fig. 3:**
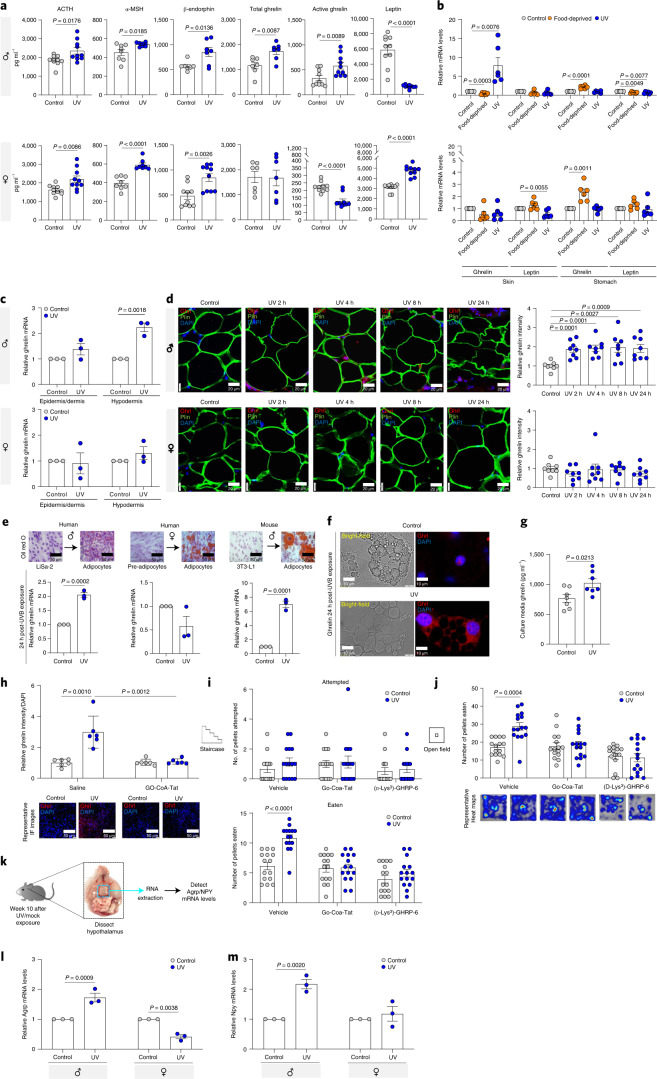
UVB radiation induces ghrelin production and secretion in skin adipocytes. **a**, Plasma levels of indicated proteins upon UVB (50 mJ/cm^2^) or mock-UVB (control) radiation, 10 weeks post first treatment (ACTH, active ghrelin and leptin: *n* = 10 biologically independent mice per condition; α-MSH: *n* = 8 biologically independent mice per condition; Total ghrelin: *n* = 7 biologically independent mice per condition; β-endorphin: *n* = 7 biologically independent mice in each condition for males and *n* = 10 biologically independent female mice per condition). **b**, Relative *ghrelin* and *leptin* mRNAs levels from stomach and skin tissues of control, UVB (50 mJ/cm^2^) irradiated, or 22-h-food-deprived mice, 5 weeks after the first UVB exposure (*n* = 5 biologically independent mice per condition). Data normalized to *36b4*. **c**, *ghrelin* mRNA levels upon 5 days daily UVB- (500 mJ/cm^2^) or mock-UVB (control) irradiated human skin (*n* = 3 biologically independent human donors per condition). Data normalized to *36b4*. **d**, Immunofluorescence analyses of ghrelin (red), perilipin 1 (Plin1, an adipocyte marker, green) and nuclei (DAPI, blue) in human skin adipose tissue at the indicated time points after a single UVB (2,000 mJ/cm^2^) or control exposure. Graphs are ghrelin fluorescence intensity normalized to DAPI (*n* = 8 fields from 3 biologically independent human donors). **e**, Indicated cell lines differentiated into mature adipocytes (validated with Oil Red O staining). Bottom panel: Relative *ghrelin* mRNA levels in UVB-irradiated (50 mJ/cm^2^) or control indicated cells, 24 h after the treatment (*n* = 3 biologically independent samples per condition). Data normalized to *18S* or *36b4*. **f**, Immunofluorescence of ghrelin (red) and nuclei (DAPI, blue) in differentiated 3T3-L1 adipocytes from (e). **g**, Secreted ghrelin levels from differentiated 3T3-L1 adipocytes from (e) (*n* = 8 biologically independent samples per condition). **h**, Differentiated 3T3-L1 adipocytes treated with ghrelin inhibitor (GO-CoA-Tat 6 µM) or saline for 3–4 h before treatment and immunofluorescence analysis as in (f) (*n* = 6 fields from 3 biologically independent samples for each condition). **i**, Staircase test analysis for male mice 8 weeks after initiation of daily UVB (50 mJ/cm^2^) or mock-UVB (control), treated with ghrelin inhibitor (GO-CoA-Tat (192 µg/Kg)), Ghrelin-receptor antagonist ([d-Lys^3^]-GHRP-6 (200 nmol/mouse)) or saline, 1–2 h before the staircase test (*n* = 15 biologically independent mice per condition). **j**, Upper panel: Open-field analysis for male mice treated as in (i) (*n* = 15 biologically independent mice per condition). **k**, Experimental design. **l**,**m**, Relative *Agrp* (l) and *Npy* (m) mRNA levels in the hypothalamus upon indicated treatments (*n* = 3 biologically independent mice per condition). Data normalized to *36b4*. In all relevant panels: Data are presented as mean ± SEM; Two-tailed unpaired *t*-test *p*-values are shown, or statistical details for sex or UVB or treatment factors in the ANOVAs (*F*-values, degrees of freedom, *p*-value) with interaction appears in Supplementary Table 10, or two-way ANOVA analysis with multiple correction test appears in Supplementary Table 11. Source data

UVB exposure of the skin activates p53 and its downstream multicomponent precursor polypeptide POMC, which is cleaved to yield α-MSH (melanocyte-stimulating hormone)^31^, adrenocorticotropic hormone (ACTH) and β-endorphin^7^. All three POMC derivatives have the potential to be released into the blood, but it was shown only for β-endorphin^7^. We found that, upon daily UVB exposure, ACTH, α-MSH and β-endorphin were significantly increased in the plasma of both males and females (Fig. 3a). This strengthens our hypothesis that the increase in food intake and food-seeking behavior by males exposed to UVB cannot be explained by an increase in β-endorphin but rather by an additional unknown factor.

**Extended Data Fig. 4:**
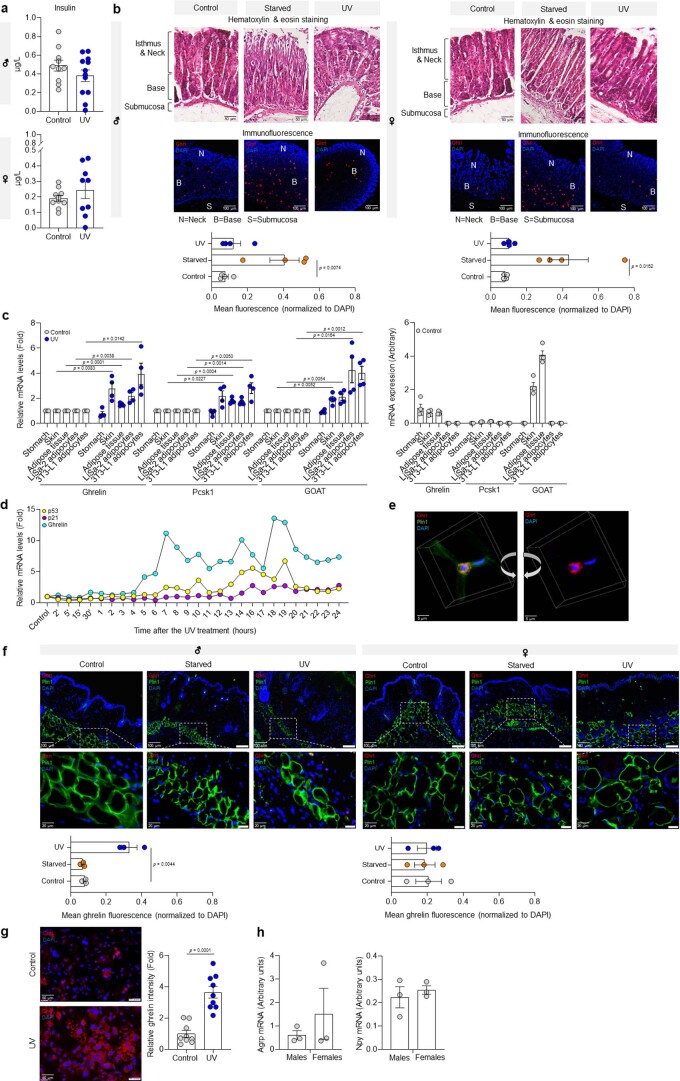
UVB radiation induces ghrelin production and secretion in skin adipocytes, Related to Fig. 3. **a**, Plasma protein levels of insulin upon UVB (50 mJ/cm^2^) or mock-UVB (control) -irradiated mice at 10 weeks after the first treatment (Biologically independent males: *n* = 10 control and *n* = 12 UV biologically independent mice per condition; Biologically independent females: *n* = 10 control and *n* = 9 UV mice per condition). **b**, Stomach tissue from control, UVB-irradiated (50 mJ/cm^2^), and 22-h-food-deprived mice after 5 weeks of treatment. Immunofluorescence for ghrelin (red), perilipin 1 (Plin1, an adipocyte marker, green), and nuclei (DAPI, blue). Ghrelin intensity normalized to DAPI (*n* = 4 biologically independent mice per condition). **c**, Relative and arbitrary mRNA levels of *pcsk1*, *GOAT*, and *ghrelin* from indicated tissue from UVB (50 mJ/cm^2^) or mock-UVB (control) irradiated male mice at 10 weeks after first treatment and from LiSa-2 differentiated adipocytes 24 h after UVB (50 mJ/cm^2^) radiation. Data normalized to *36b4* (*n* = 4 biological replicates). **d**, Relative *p53* and *p21* mRNA levels from differentiated 3T3-L1 adipocytes 24 h after UVB (50 mJ/cm^2^) or control radiation. Data normalized to *36b4*. **e**, Representative 3D construction of immunofluorescence image of human skin adipose tissue of UVB (2,000 mJ/cm^2^) 24 h post-treatment stained for ghrelin (red), perilipin 1 (Plin1, an adipocyte marker, green), and nuclei (DAPI, blue). **f**, Skin tissue from (b). Upper panel: Representative images of immunofluorescence. Bottom panel: Ghrelin intensity normalized to DAPI (*n* = 3 biologically independent mice per condition). **g**, Left panel: Immunofluorescence of differentiated 3T3-L1 adipocytes after 24 h of UVB (50 mJ/cm^2^) or control treatments stained for ghrelin (red) and nuclei (with DAPI, blue). Right panel: Ghrelin intensity normalized to DAPI intensity (*n* = 9 random fields from 3 biological replicates). **h**, Arbitrary of *Agrp* and *Npy* mRNA levels from brain hypothalamus upon 10 weeks of mock-UVB (control) treatment (*n* = 3 biologically independent mice per condition). Data normalized to *36b4*. In all relevant panels: Data are presented as mean ± SEM; Two-tailed unpaired *t*-test *p*-values are shown, or statistical details for sex or UVB or treatment factors in the ANOVAs (*F*-values, degrees of freedom, *p*-value) with interaction appears in Supplementary Table 10, or two-way ANOVA analysis with multiple correction test appears in Supplementary Table 11. Source data

Further analysis of the plasma of post-UVB exposed mice revealed a significant increase in the level of ghrelin in males, both in its total form or active form (acyl ghrelin) but not in females (Fig. 3a). Conversely, the levels of leptin were significantly lower upon males’ exposure to UVB (Fig. 3a) while in females, leptin levels significantly increased (Fig. 3a). Further, since insulin is involved in food intake regulation^32^, we found no change in concentration of circulating insulin in male or female mice upon UVB exposure (Extended Data Fig. 4a).

The stomach is the main source of ghrelin^8^ and white adipose tissue is the major site of leptin^22^. Although the skin’s involvement in food-seeking behavior regulation is unknown, it does include white adipose in the hypodermis^6^, so we investigated skin and stomach tissues as potential producers of appetite-related hormones upon UVB exposure. After food deprivation, *ghrelin* mRNA and protein levels were significantly higher in the stomachs of both males and females compared to mock-UVB-treated mice (Fig. 3b, Extended Data Fig. 4b). Conversely, UVB exposure (50 mJ/cm^2^) significantly increased *ghrelin* levels and those of enzymes involved in its biogenesis, that is, ghrelin-O-acyltransferase (GOAT)^32^ and pro-hormone convertase (*Pcsk1*) 1/3^33^, in the skin but not in the stomach of males (Fig. 3b, Extended Data Fig. 4c). UVB exposure had no effect on *ghrelin* levels in female skin (Fig. 3b, Extended Data Fig. 4f). Upon UVB exposure, *Leptin* expression levels slightly decreased in male skin, significantly decreased in male stomach, significantly decreased in female skin and slightly decreased in female stomach (Fig. 3b). Since there was a significant increase in plasma leptin levels in females after UVB exposure, another organ must be involved in leptin regulation upon UVB exposure in female mice.

Next, we exposed ex vivo human skin to daily (five days) UVB radiation (500 mJ/cm^2^) or mock-UVB radiation and found that *ghrelin* mRNA level in the males hypodermis adipose tissue was significantly higher upon UVB exposure (Fig. 3c). In contrast, *ghrelin* expression in female skin hypodermis did not alter upon UVB exposure (Fig. 3c). The epidermal and dermal layers of the human skin from males and females showed no change in *ghrelin* levels upon UVB treatment (Fig. 3c). A time-course experiment revealed a peak in ghrelin protein expression 8 h post UVB exposure in male, but not female, hypodermal adipocytes (Fig. 3d), in the nucleus (Extended Data Fig. 4e, Supplementary Video 1). This illustrates that ghrelin is induced in male hypodermal skin adipocytes after exposure to UVB.

White adipose tissue (primarily located beneath the skin but also around organs, especially in the abdominal cavity) secretes factors such as adipokines, interferons, interleukins and growth factors, exerting a wide range of biological actions such as inflammation, regulation of food intake and insulin sensitivity^34^. To confirm adipocyte function upon UVB exposure, we differentiated human male LiSa-2 cells, primary human female pre-adipocytes, and mouse male 3T3-L1 cells into adipocyte-like cells, as validated by Oil Red O staining for lipid droplets (Fig. 3e). UVB exposure (50 mJ/cm^2^) led to significant increases in *ghrelin,* *GOAT*, and *Pcsk1* mRNA levels compared to mock-UVB-treated cells (Fig. 3e, Extended Data Fig. 4d,e). UVB treatment had no effect on ghrelin expression in human female adipocytes. Ghrelin protein secretion, too, was elevated in the cell culture medium upon 50 mJ/cm^2^ UVB (Fig. 3f,g and Extended Data Fig. 4g). The addition of ghrelin O-acyltransferase (GOAT) inhibitor GO-CoA-Tat^32^ diminished the UV effect on ghrelin production compared to vehicle- and UVB-treated cells (Fig. 3h).

Investigation of food-seeking behavior, we found that UVB effect was abrogated in mice treated with the GO-CoA-Tat and in mice treated with the ghrelin receptor antagonist [D-Lys^3^]-GHRP-6 (ref. ^32^) (Fig. 3i). Neither of the drugs had a significant effect on eating behavior of the mock-UVB-treated animals (Fig. 3i). Moreover, the attempt score did not change upon UVB, GO-CoA-Tat, or [D-Lys^3^]-GHRP-6 treatments, indicating no effect on motor functions (Fig. 3i).

In the open-field test, males treated with both vehicle and UVB ate significantly more food pellets than vehicle- and mock-UVB-treated males (Fig. 3j). This effect was abolished in UVB-treated males treated with GO-CoA-Tat or [d-Lys^3^]-GHRP-6 (Fig. 3j). These results demonstrate that UVB exposure increases food craving in males via ghrelin induction. The orexigenic role of ghrelin in promotion of food intake in the fasting state is dependent on activity of neurons that express AgRP and NPY^32^ from brain hypothalamus (Fig. 3k). Both *Agrp* and *Npy* mRNA levels are significantly higher in UVB-exposed male mice, whereas UVB treatment did not alter these mRNA levels in female mice (Fig. 3l,m, Extended Data Fig. 4h), which is in correlation with the increased active ghrelin concentrations in plasma. Taken together, our data demonstrate that UVB radiation induces ghrelin production and secretion from male skin adipocytes but not from female skin adipocytes and that ghrelin mediates the effects of UVB on food-seeking behavior of male mice.

### p53 regulates UVB-induced ghrelin expression

**Extended Data Fig. 5:**
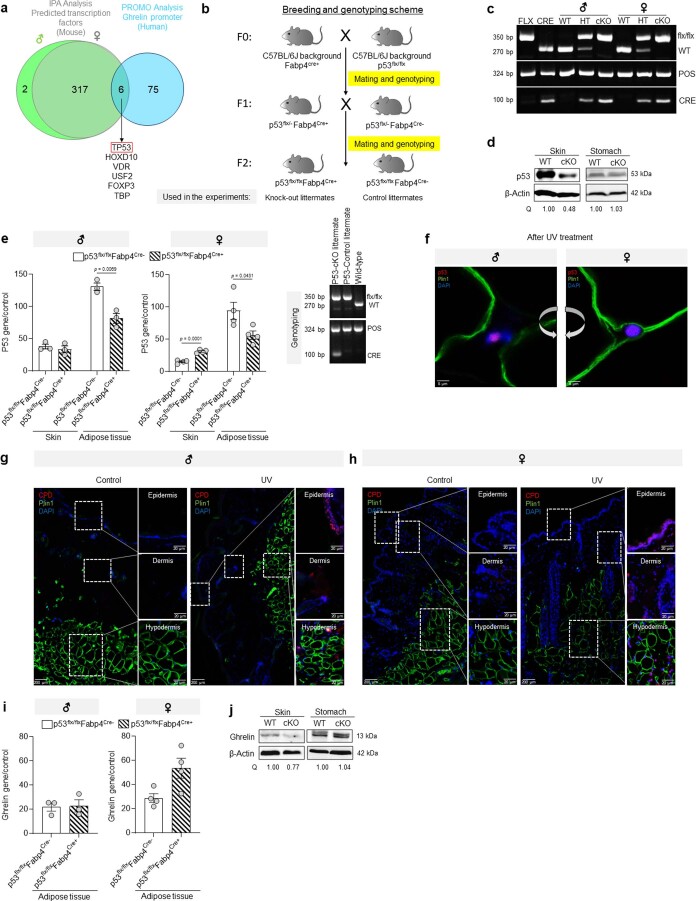
p53 regulates UVB induced *ghrelin* expression, Related to Fig. 4. **a**, Venn diagram of transcription factors identified using Ingenuity Pathway analysis from male and female mouse plasma proteomics (UVB/control) and putative transcription factors that bind sites identified in the human *ghrelin* promoter using PROMO. **b**, Experimental design. **c**, Flox and Cre expression analysis of genomic DNA from the WAT. **d**, p53 protein levels from skin and stomach tissues of mock-UVB (control) irradiated wild-type C57BL/6 J (WT) and p53-cKO male mice 10 weeks after the first treatment. β-actin was used as the loading control. Quantification of protein amount normalized to β-actin (Q) is indicated. **e**, Baseline levels of *p53* mRNA in wild-type and p53-cKO mice in skin and adipose tissue (*n* = 3 biologically independent male and *n* = 4 biologically independent female mice per condition). Data normalized to *36b4*. **f**, Representative 3D reconstructed image of immunofluorescence image of men (left panel) and women (right panel) skin adipose tissue 24 h after UVB (2,000 mJ/cm^2^); the tissue was stained post-treatment for p53 (red), perilipin 1 (Plin1, an adipocyte marker, green), and nuclei (DAPI, blue). **g**,**h**, Immunofluorescence analysis of skin tissue from (**g**) male and (**h**) female p53-cKO (p53^flx/flx^Fabp4^Cre+^) mice after 10 weeks of daily UVB (50 mJ/cm^2^) or control irradiation. The skin layers (epidermis, dermis, and hypodermis) were stained for cyclobutane pyrimidine dimers (red), perilipin 1 (Plin1, an adipocyte marker, green), and nuclei (with DAPI, blue). **i**, Baseline levels of *ghrelin* mRNA in control and p53-cKO mice (*n* = 3 biologically independent male and *n* = 4 biologically independent female mice per condition). *Ghrelin* levels were normalized to *36b4*. **j**, Western blot analysis of ghrelin protein levels from skin and stomach tissues of mock-UVB (control) irradiated wild-type C57BL/6 J (WT) and p53-cKO male mice 10 weeks after the first treatment. β-actin was used as the loading control. Quantification of the protein amount normalized to β-actin (Q) is indicated. In all relevant panels: Data are presented as mean ± SEM; Two-tailed unpaired *t*-test *p*-values are shown, or statistical details for sex or UVB or p53-cKO factors in the ANOVAs (*F*-values, degrees of freedom, *p*-value) with interaction appears in Supplementary Table 10, or two-way ANOVA analysis with multiple correction test appears in Supplementary Table 11. Source data

Our finding that ghrelin induction peaks 8 h after UVB exposure suggests transcriptional regulation. We conducted an Ingenuity Pathway Analysis of the group of plasma proteins that showed differential expression upon daily UVB exposure (Fig. 1e) in an effort to predict possible upstream regulators (Supplementary Table 3). We further analyzed the sequence of the ghrelin promoter for predicted transcription factor binding sites using PROMO (Supplementary Table 4). Crossing these lists yielded six potential candidates: p53, HOXD10, VDR, USF2, FOXP3 and TBP (Extended Data Fig. 5a). p53 was the most significant among these factors (Extended Data Fig. 5a) and since UV-induced DNA damage directly activates p53^35^, we reasoned that UVB-induced ghrelin expression in adipocytes might be mediated by p53.

**Fig. 4:**
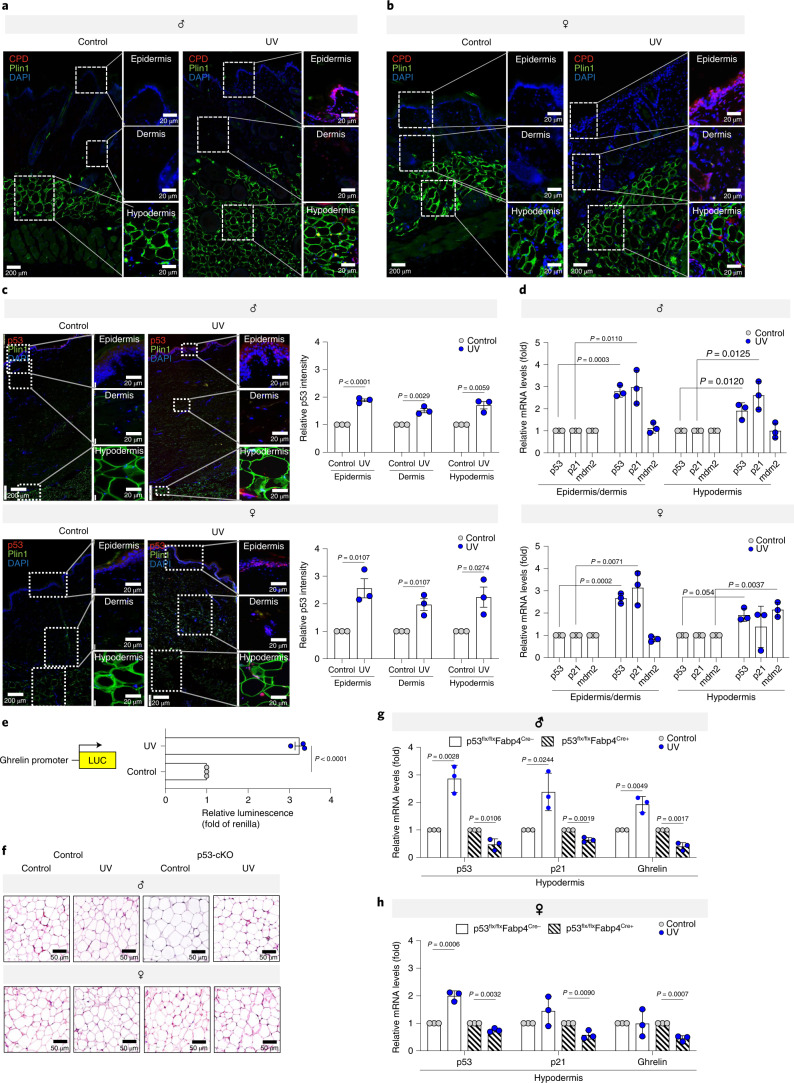
p53 regulates UVB-induced *ghrelin* expression. **a**,**b**, Immunofluorescence analysis of the skin of control (**a**) male and (**b**) female mice (p53^flx/flx^Fabp4^Cre-^) after 10 weeks of daily UVB (50 mJ/cm^2^) or control irradiation. Indicated skin layers stained for cyclobutane pyrimidine dimers (red), perilipin 1 (Plin1, an adipocyte marker, green) and nuclei (with DAPI, blue). **c**, Immunofluorescence analysis of human skin tissue at 24 h after either UVB (2,000 mJ/cm^2^) or mock-UVB (control) irradiation. p53 intensity was normalized to DAPI intensity. (*n* = 3 biologically independent human donors per condition). Representative images of male (left) and female (right) skin layers. **d**, mRNA levels of *p53*, *p21* and *mdm2* in the epidermis/dermis and hypodermis of indicated human skin tissue upon 5 days of either UVB (500 mJ/cm^2^/day) or control radiation (*n* = 3 biologically independent human donors per condition). Data normalized to *36b4*. **e**, Luciferase activity downstream to the human *ghrelin* promoter (−3,000 bp upstream) in the presence of p53 or an empty vector (control) in H1299 cells 48 h after transfection. Firefly luciferase activity normalized to *Renilla* luciferase activity (*n* = 3 biologically independent samples per condition). **f**, H & E staining of adipose tissue from control (p53^flx/flx^Fabp4^Cre-^) and p53-cKO (p53^flx/flx^Fabp4^Cre+^) male and female mice after 10 weeks of daily exposure to UVB (50 mJ/cm^2^) or mock-UVB (control) irradiation. **g**, Relative *p53*, *p21* mRNA levels and *ghrelin* from the hypodermis of dorsal skin of control (p53^flx/flx^Fabp4^Cre-^) and p53-cKO (p53^flx/flx^Fabp4^Cre+^) male mice after 10 weeks of daily UVB (50 mJ/cm^2^/day) or mock-UVB (control) irradiation (*n* = 3 biologically independent mice per condition). Data normalized to *36b4*. **h**, Relative *p53*, *p21* and *ghrelin* mRNA levels in the hypodermis of dorsal skin of control and p53-cKO female mice after 10 weeks of daily UVB (50 mJ/cm^2^/day) or mock-UVB (control) irradiation (*n* = 3 biologically independent mice per condition). Data normalized to *36b4*. In all relevant panels: Data are presented as mean ± SEM; Two-tailed unpaired *t*-test *p*-values are shown and statistical details for sex or UVB or p53-cKO factors in the ANOVAs (*F*-values, degrees of freedom, *p*-value) with interaction appears in Supplementary Table 10. Source data

Can UVB radiation penetrate the hypodermis? UVB induces DNA damage mainly at dipyrimidine sites, resulting in the formation of cyclobutane pyrimidine dimers (CPDs) and the pyrimidine-pyrimidone (6-4) photoproduct (6-4PP)^30^. We detected CPDs in the epidermis, dermis, and hypodermis of UVB-irradiated male and female mice skin but not in that of mock-UVB-treated mice (Fig. 4a,b). Confirmation that UV exposure induces p53 activity was obtained by exposing explanted human skin to either UVB (2,000 mJ/cm^2^) or mock-UVB irradiation, which led to the significant upregulation of p53 at the protein level in all three skin layers (epidermis, dermis, and hypodermis) in both males and females (Fig. 4c) nucleus (Extended Data Fig. 5f, Supplementary Video 2, 3) upon UVB exposure. Both p53 and its downstream target p21 were upregulated in the epidermis/dermis and the hypodermis of males and females upon UVB exposure, at the mRNA level (Fig. 4d). The increase in p53 upon UVB was not due to decreased expression of its known negative regulator Mdm2^36^ (Fig. 4d).

To ascertain whether p53 directly induces the transcription of *ghrelin*, we co-transfected a luciferase reporter driven by the *ghrelin* promoter^13^ with a p53 expression vector. The reporter’s activity was significantly induced by the presence of p53 (Fig. 4e), suggesting that *ghrelin* is a direct transcriptional target of p53.

We next crossed mice that express Cre specifically in white adipose tissue (under the *Fabp4* promoter) with *p53*-floxed mice, to generate mice with conditional knockout (cKO) of p53 in adipocytes (p53^flx/flx^Fabp4^Cre-^) (Extended Data Fig. 5b,c). p53 protein level was specifically reduced in skin, while stomach p53 levels were not altered by p53-cKO (Extended Data Fig. 5d). *p53* mRNA level was significantly decreased in mouse skin hypodermis compared to the epidermis/dermis (Extended Data Fig. 5e) for male and female mice. There were no phenotypic changes in the adipose tissue upon *p53* depletion (Fig. 4f) and DNA damage was observed in skin adipocytes of the p53-cKO mice following UVB exposure (Extended Data Fig. 5g,h). Upon UVB exposure (50 mJ/cm^2^), the hypodermis expression of *p53* and *p21*, was heightened in control mice (Fig. 4g), while in p53-cKO mice it was not elevated (Fig. 4g). In the same exposure experiment, we noted an elevation in *ghrelin* levels in the hypodermis of the control males, but *a* significant decrease in p53-cKO males (Fig. 4g). In the female mice groups exposed to UVB light, *ghrelin* levels did not alter in the hypodermis of wild-type females, whereas in p53-cKO females there was a significant drop in *ghrelin* levels (Fig. 4h). The basal level of *ghrelin* mRNA was similar in the control littermates and p53-cKO mice (Extended Data Fig. 5i) but at protein level ghrelin showed significant drop in skin and not stomach of p53-cKO mice compared to control (Extended Data Fig. 5j). Taken together, our data demonstrate that p53 directly upregulates *ghrelin* expression in male, but not in female, skin adipose tissue in response to UVB light.

### Deletion of *p53* in skin adipocytes abrogates UVB-induced food-seeking behavior

**Fig. 5:**
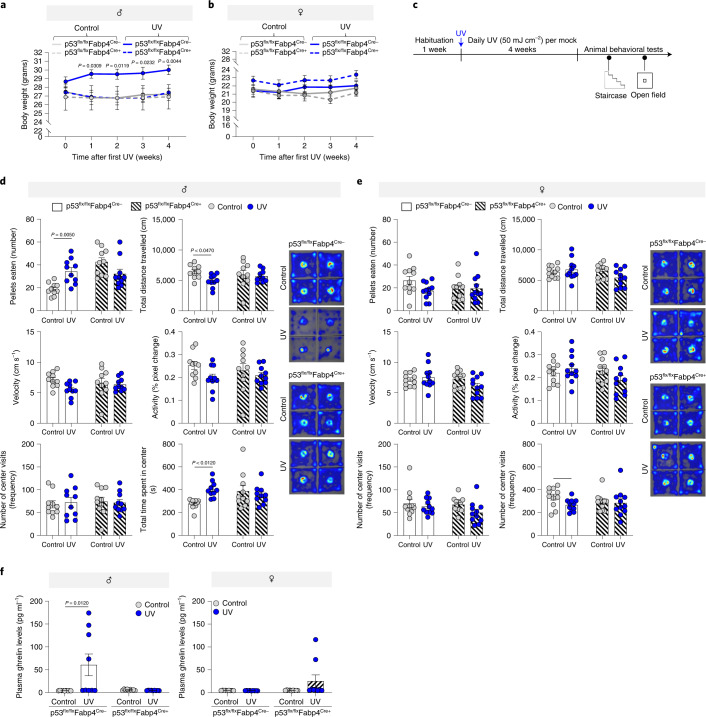
Deletion of p53 in skin adipocytes abrogates UVB-induced appetite enhancement. **a**,**b**, Relative weekly body weight of male (**a**) and female (**b**) mice after 4 weeks of daily UVB (50 mJ/cm^2^) or control irradiation (Males: *n* = 10 biologically independent p53^flx/flx^Fabp4^Cre+^ mice per condition and *n* = 7 biologically independent p53^flx/flx^Fabp4^Cre-^ mice per condition; Females: *n* = 9 biologically independent p53^flx/flx^Fabp4^Cre+^ mice per condition and *n* = 11 biologically independent p53^flx/flx^Fabp4^Cre-^ mice per condition). **c**, Experimental design. **d**,**e**, Responses of control and p53-cKO (**d**) male and (**e**) female mice in the open-field test after 5 weeks of exposure to UVB (50 mJ/cm^2^) or mock-UVB (control) irradiation (*n* = 10 biologically independent male mice per condition and *n* = 11 biologically independent female mice per condition). Right panel: Representative heat map images from the video of the test session. **f**, Plasma protein levels of ghrelin in UVB (50 mJ/cm^2^) or mock-UVB (control)-irradiated control and p53-cKO male (left panel) and female (right panel) mice after 5 weeks of treatment (*n* = 9 biologically independent mice per condition). In all relevant panels: Data are presented as mean ± SEM; Two-tailed unpaired *t*-test *p*-values are shown, or statistical details for sex or UVB or p53-cKO factors in the ANOVAs (*F*-values, degrees of freedom, *p*-value) with interaction appears in Supplementary Table 10, or two-way ANOVA analysis with multiple correction test appears in Supplementary Table 11. Source data

**Extended Data Fig. 6:**
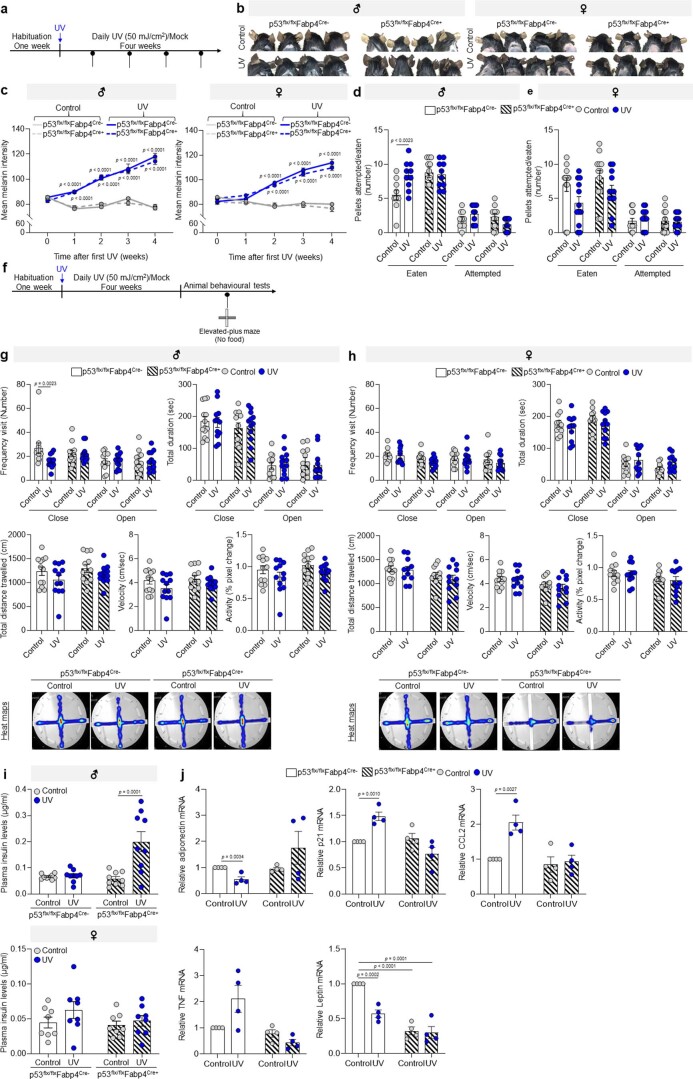
Deletion of p53 in skin adipocytes abrogates UVB-induced appetite enhancement, Related to Fig. 5. **a**, Experimental design. **b**, Melanin pigment accumulation in the mice ears upon indicated treatment (a). **c**, Weekly mean ears melanin levels of indicated mice upon indicated treatment. (Biologically independent Males: *n* = 8 mice per condition; Biologically independent females: *n* = 7 p53^flx/flx^Fabp4^Cre-^ control/UV, p53^flx/flx^Fabp4^Cre+^ control and *n* = 8 p53^flx/flx^Fabp4^Cre+^ UV). **d**,**e**, Staircase test for control and p53-cKO (**d**) male (*n* = 10 biologically independent mice per condition) and (**e**) female mice after 5 weeks of daily UVB (50 mJ/cm^2^) or mock-UVB (control) irradiation. Shown are the measured number of sucrose pellets eaten and of attempts to reach a pellet. (*n* = 12 biologically independent mice per condition). **f**, Experimental design. **g**,**h**, Upper & middle panels: Number of visits to open and closed arms during 5 min; duration of time spent in the open or closed arms, total distance travelled; velocity; and activity of control and p53-cKO (**g**) male (*n* = 12 biologically independent mice per condition) and (**h**) female mice after 4 weeks of daily UVB (50 mJ/cm^2^) or mock-UVB (control) irradiation. Lower panel: A representative image of the heat map of the maze for each condition. (*n* = 11 biologically independent mice per condition). **i**, Plasma insulin levels in p53-cKO mice after 5 weeks of UVB (50 mJ/cm^2^) or mock-UVB (control) irradiated control treatment. (*n* = 9 biologically independent mice per condition). **j**, Relative mRNA levels of Adiponectin, *p21*, *Ccl2*, *Tnf*, and *leptin* in the skin adipose tissue of control and p53-cKO male mice after 5 weeks of daily UVB (50 mJ/cm^2^) or mock-UVB (control) exposure (*n* = 4 biologically independent mice per condition). Data normalized to *36b4*. In all relevant panels: Data are presented as mean ± SEM; Two-tailed unpaired t-test *p*-values are shown, or statistical details for sex or UVB or p53-cKO factors in the ANOVAs (*F*-values, degrees of freedom, *p*-value) with interaction appears in Supplementary Table 10, or two-way ANOVA analysis with multiple correction test appears in Supplementary Table 11. Source data

To examine the functional requirements of p53 in UVB-induced food-seeking behavior changes, we performed the above-mentioned animal behavioral tests (Fig. 5a-e, Extended Data Fig. 6a-h) on p53-cKO mice and compared the results. Of a note, following the daily UVB treatment, the amount of pigment accumulation in the ears of mock-UVB-treated and p53-cKO animals was similar and there were no sex differences (Extended Data Fig. 6b,c), indicating that epidermal p53 is intact^31,37^. Further, chronic UVB exposure significantly induces weight gain in control (p53^flx/flx^Fabp4^Cre-^) male mice, but in cKO (p53^flx/flx^Fabp4^Cre+^) animals the UVB effect is abrogated (Fig. 5a). Body weights of female mice were stable in all the models and experimental conditions (Fig. 5b).

In the open-field test, p53-cKO males treated daily with UVB (50 mJ/cm^2^) did not differ from mock-UVB-irradiated p53-cKO controls; this differed from the effect of UVB on control mice (Fig. 5c,d). p53-cKO and control (p53^flx/flx^Fabp4^Cre^) females did not show significant differences in any of the open-field test parameters with or without UVB treatment (Fig. 5e). In the staircase test, control (p53^flx/flx^Fabp4^Cre-^) male mice treated daily with UVB (50 mJ/cm^2^) consumed more food pellets than mock-UVB-treated male mice, an affect that was abolish in the p53-cKO mice (Extended Data Fig. 6d,e). There were no notable differences in food consumption among the female colony or in the number of attempts made by any of the male groups to reach pellets (Extended Data Fig. 6d, e). No significant effect of p53 absence in adipocytes was observed in the elevated-plus maze test in either males or females upon daily UVB (50 mJ/cm^2^) treatment (Extended Data Fig. 6f-h). We noticed that p53-cKO mice consume more pellets than their control counterparts (Fig. 5d), independent of UVB exposure. p53 is suggested to be a central regulator of food intake and is positioned at the fulcrum between food-intake enhancement (β-endorphin^38^) and inhibition (leptin^32^, insulin^39^, α−MSH^40^). For example, p53 was suggested to govern insulin resistance in adipose tissue via the regulation of proinflammatory cytokines^41^, which we found to be altered in their expression upon UVB exposure (Extended Data Fig. 6j). Further, circulating insulin levels in male, but not female, p53-cKO mice were significantly elevated after chronic UVB exposure (Extended Data Fig. 6i). Therefore, we do not exclude the possibility that abolishing p53 could affect additional mediators of food intake.

Consistently, ghrelin levels were significantly higher in the blood plasma of p53^flx/flx^Fabp4^Cre-^ male mice following UVB treatment, an effect abrogated in the p53^flx/flx^Fabp4^Cre+^ counterparts (Fig. 5f). Neither p53^flx^^/flx^Fabp4^Cre-^ nor p53^flx/flx^Fabp4^Cre+^ female mice showed any significant difference (Fig. 5f). This data demonstrates that enhancement of food-seeking behavior in male mice upon UVB exposure is p53 dependent.

### Estrogen blocks p53 transcriptional activation of *ghrelin* in response to UVB

**Fig. 6:**
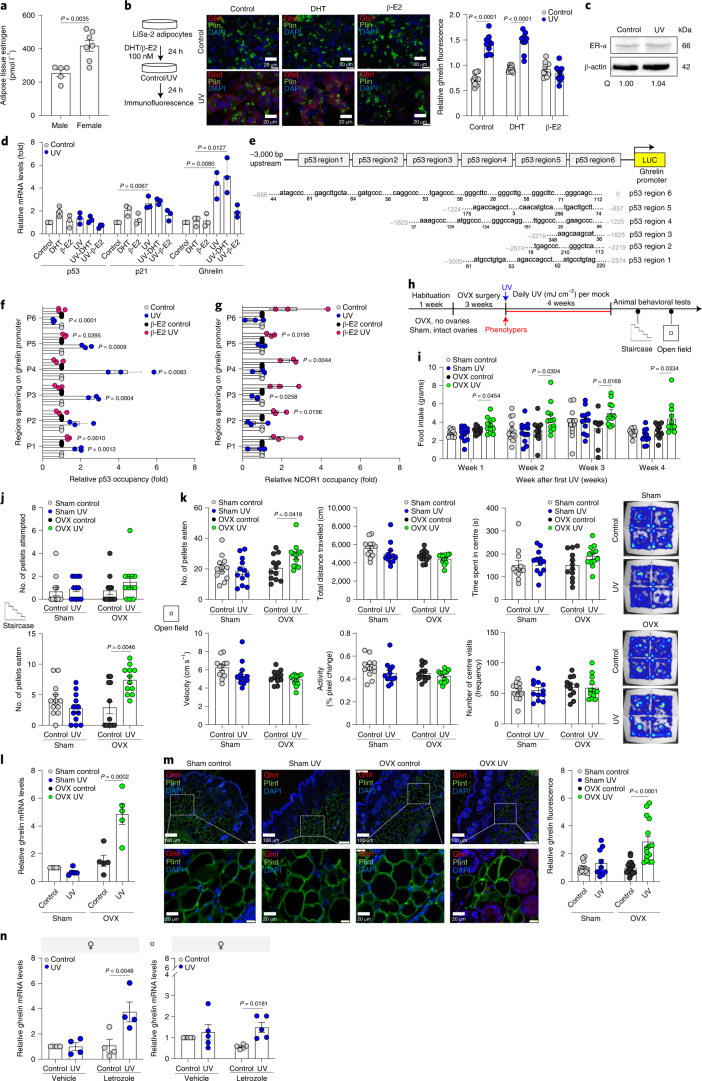
Estrogen blocks p53 transcriptional activation of ghrelin upon UVB exposure. **a**, Estrogen levels in human skin adipose tissue (*n* = 5 and *n* = 7 independent human male and female donors, respectively). **b**, Left: Experimental design. Right: Representative images of cells stained for ghrelin (red), perilipin 1 (Plin1, an adipocyte marker, green) and nuclei (with DAPI, blue) (*n* = 10 random fields from 3 biologically independent samples per condition). Relative ghrelin intensity was normalized to DAPI. **c**, ER-α protein levels in differentiated LiSa-2 adipocytes 24 h after UVB (50 mJ/cm^2^) or mock-UVB (control) irradiation treatments. β-actin was used as the loading control. Quantification of the protein amount normalized to β-actin (Q). **d**, mRNA levels of *p53*, *p21* and *ghrelin* in differentiated LiSa-2 adipocytes 24 h post treatment with 100 nM DHT or 100 nM β-E2 or vehicle and UVB (50 mJ/cm^2^) or mock-UVB (control) irradiation (*n* = 3 biologically independent samples per condition). Data normalized to *36b4*. **e**, Schematic representation of ghrelin promoter with p53 binding regions. **f**,**g**, p53 (**f**) and NCOR1 (**g**) occupancy over human *ghrelin* promoter in differentiated LiSa-2 adipocytes treated as in (c) (*n* = 3 biologically independent samples per condition). ChIP levels (fold) normalized to input. **h**, Experimental design. **i**, PhenoTyper analysis of weekly food intake (in grams) of indicated female mice as in (h) (Week 1 and 2: *n* = 12 biologically independent mice per condition; Week 3: *n* = 12 biologically independent mice per sham condition, *n* = 10 biologically independent mice for OVX control and *n* = 11 biologically independent mice for OVX UV; Week 4: *n* = 11 biologically independent mice per condition). **j**, Staircase test for indicated female mice and treatments as in (h) (*n* = 12 biologically independent mice per condition). **k**, Open-field analysis by the indicated mice and treatments (h) (*n* = biologically independent 12 mice per condition). Right panel: Representative heat maps. **l**, Relative *ghrelin* mRNA levels in OVX or sham female mice skin, after 5 weeks of daily UVB (50 mJ/cm^2^) or mock-UVB (control) exposures (*n* = 5 biologically independent mice per condition). Data normalized to *36b4*. **m**, Upper panel: Representative immunofluorescence images stained as in (b), in skin tissue from (l). Ghrelin intensity normalized to DAPI (*n* = 10 images from 3 biologically independent mice per condition). **n**, Relative *ghrelin* mRNA levels in human female skin adipose tissue 25 h post treatment with estrogen inhibitor (leterozole (5 µM)) or vehicle (DMSO) and a single UVB (500 mJ/cm^2^) or mock-UVB (control) irradiation session (*n* = 4 biologically independent human donors per condition). Data normalized to *36b4*. **o**, Relative *ghrelin* mRNA levels in differentiated primary human female adipocytes after treatment as in (n) (*n* = 5 biologically independent samples per condition). Data normalized to *36b4*. In all relevant panels: Data are presented as mean ± SEM; Two-tailed unpaired *t*-test *p*-values are shown, or statistical details for OVX or UVB factors in the ANOVAs (*F*-values, degrees of freedom, *p*-value) with interaction appears in Supplementary Table 10, or two-way ANOVA analysis with multiple correction test appears in Supplementary Table 11. Source data

**Extended Data Fig. 7:**
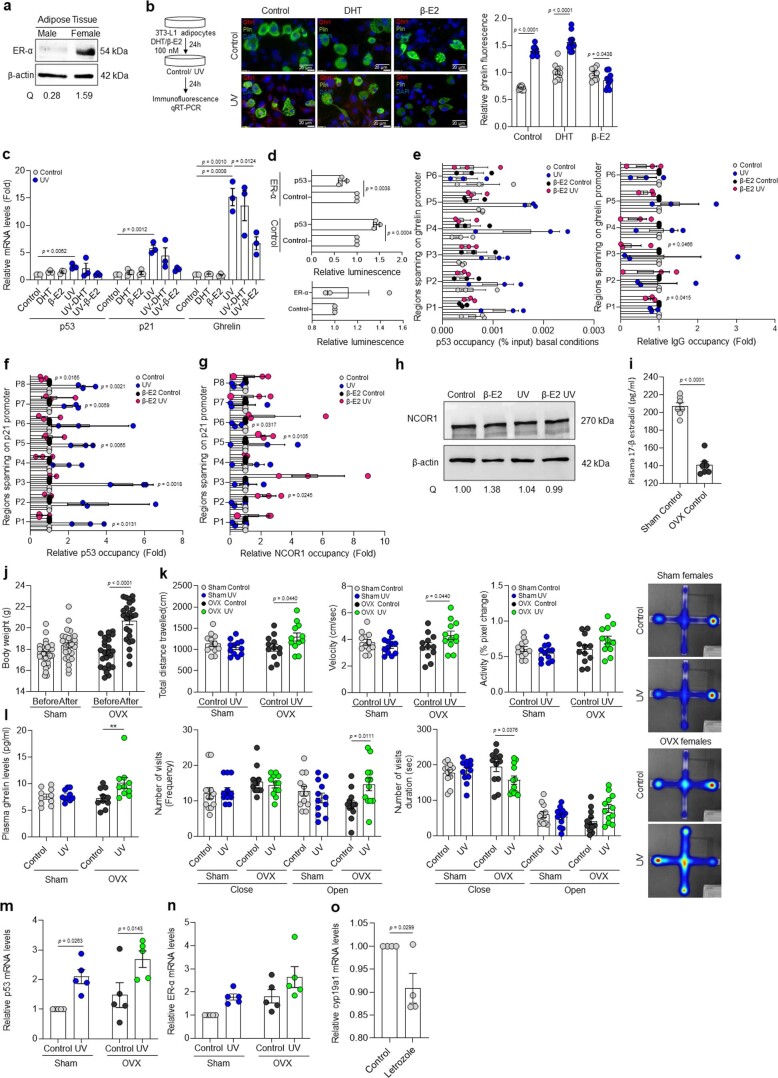
Estrogen blocks p53 transcriptional activation of ghrelin upon UVB, Related to Fig. 6. **a**, ER-α protein levels in human adipose tissue. β-actin used as the loading control. Quantification of the protein amount normalized to β-actin (Q) is indicated. **b**, Left panel: Experimental design. Right panel: Immunofluorescence of cells stained for ghrelin (red), perilipin 1 (Plin1, an adipocyte marker, green) and nuclei (with DAPI, blue) (*n* = 10 random fields from 3 biological replicates). Relative ghrelin intensity normalized to DAPI. **c**, *p53*, *p21* and *ghrelin* relative mRNA levels in 3T3-L1 differentiated adipocytes treated with a vehicle, 100 nM DHT or 100 nM β-E2 and with UVB (50 mJ/cm^2^) or control irradiation after 24 h (*n* = 3 biological replicates). Data normalized to *18S*. **d**, Upper panel: Luciferase activity upstream of the human *ghrelin* promoter (−3,000 bp upstream) in the presence of ER-α or p53, or an empty vector (control) in HeLa cells with or without 100 nM β-E2 treatment for 24 h. Lower panel: Luciferase activity similar as in upper panel in H1299 cells upon ER-α or an empty vector (control) expression. Firefly luciferase activity normalized to *Renilla* luciferase activity (*n* = 3 biological replicates). **e**–**g**, ChIP levels (fold) normalized to input of p53 and IgG occupancy over ghrelin promotor (**e**); p53 (**f**) and NCOR1 (**g**) occupancy over p21 promotor in LiSa-2 adipocytes treated with 100 nM β-E2 or vehicle and irradiated with UVB (50 mJ/cm^2^) or mock-UVB (control) and harvested after 24 h (*n* = 3 biological replicates). **h**, NCOR1 protein level in LiSa-2 adipocytes treated as in (e). β-actin was used as the loading control. Quantification of the protein amount normalized to β-actin (Q) is indicated. **i**, 17-β estradiol plasma protein levels in OVX or sham female mice after 5 weeks of either daily UVB (50 mJ/cm^2^) or mock-UVB (control) exposures (*n* = 7 biologically independent mice per condition). **j**, Weekly mean body weight (grams) of the OVX/sham female mice after daily UVB (50 mJ/cm^2^) or mock-UVB (control) irradiation before and 3 weeks after OVX/sham surgery (*n* = 29 sham before/after and *n* = 25 OVX before/after biologically independent female mice). **k**, Elevated-plus maze (EPM) responses in female OVX/sham mice after 4 weeks of either daily UVB (50 mJ/cm^2^) or mock-UVB (control) exposures. Right panel: Representative heat maps from each condition (*n* = 10 biologically independent mice per condition). **l**, ghrelin plasma protein levels of in OVX or sham female mice after 5 weeks of daily UVB (50 mJ/cm^2^) or mock-UVB (control) exposures (*n* = 10 biologically independent mice per condition). **m**, *p53* relative mRNA levels from the skin of OVX or sham female mice treated as in (l) (*n* = 5 biologically independent mice per condition). Data normalized to *36b4*. **n**, *ER-α* relative mRNA levels from the skin of OVX or sham female mice treated as in (l) (*n* = 5 mice per condition). Data normalized to *36b4*. **o**, *Aromatase* relative mRNA levels in human female skin adipose tissue 26 h after treatment with estrogen inhibitor (leterozole (5 µM)) or vehicle (DMSO). Data normalized to *36b4*. For all relevant panels: Data are presented as mean ± SEM; an unpaired *t*-test *p*-values are shown, or statistical details for OVX or UVB factors in the ANOVAs (*F*-values, degrees of freedom, *p*-value) with interaction appears in Supplementary Table 10, or two-way ANOVA analysis with multiple correction test appears in Supplementary Table 11. Source data

Why was the increase in *ghrelin* expression and food-seeking behavior upon UVB exposure in male mice and food intake in human men not observed in females? We found testosterone and estrogen as potential regulators of the UVB effect in both males and females (Supplementary Table 5). The crosstalk between ghrelin and sex steroids has been shown before, but the mechanism has not been fully elucidated. In males, ghrelin levels positively correlate with testosterone levels^42^, whereas in females, estradiol suppresses the orexigenic effect of ghrelin^43^ in adipose tissue and decreases lipolysis^44^. Indeed, we found that the levels of estrogen and its cognate receptor ER-α are significantly higher in females than in males, in human skin explants adipose tissue (Fig. 6a, Extended Data Fig. 7a), suggesting that 17β-estradiol (β-E2) levels can explain the differences in ghrelin expression (Fig. 3c).

To evaluate the effect of sex steroids on ghrelin expression in UVB-exposed differentiated adipocytes (LiSa-2 and 3T3-L1), we stimulated the cells with either dihydro-testosterone (DHT) or β-E2, followed by UVB (50 mJ/cm^2^) or mock-UVB irradiation. DHT significantly induced *ghrelin* expression at the protein level, whereas the presence of β-E2 inhibited UVB-induced *ghrelin* expression at the protein (Fig. 6b, Extended Data Fig. 7b) and mRNA (Fig. 6d, Extended Data Fig. 7c) levels. Notably, LiSa-2 adipocytes are from male source and we found that estrogen receptor alpha (ER-α) protein levels did not change upon UVB treatment (Fig. 6c). These results are in accordance with previous literature that demonstrated that subcutaneous white adipose tissue of human males and females expressed similar amount of ER-α^45^. This explains why LiSa-2 adipocytes have the ability to respond to β-E2 treatment. Further, the ability of p53 to activate the expression of *ghrelin* promoter was significantly reduced in the presence of estrogen (Extended Data Fig. 7d). Taken together, our data demonstrate that estrogen blocks p53-dependent ghrelin induction upon UVB exposure.

ER-α binds directly to p53 and represses its transcriptional activity, affecting the expression of *p21*^46^ and *survivin*^47^ genes. Further, ER-α modulates p53 transcriptional activity through a direct interaction^48^ to the *p21* promoter^49^, following ionizing radiation^48^. By chromatin immunoprecipitation (ChIP) analysis, we found significant increase of p53 occupancy over the *ghrelin* upstream region (regions 1,3,4 and 5) and *p21*, upon UVB exposure, compared to vehicle-treated cells, whereas the presence of estrogen blocked p53 recruitment to the *ghrelin* promotor (Fig. 6e and Extended Data Fig. 7e). Additionally, we found significant abrogation in recruitment of p53 in the presence of β-E2 upon UVB exposure compared to UVB exposure in the absence of β-E2 to both *ghrelin* and *p21* promotors (Fig. 6f, Extended Data Fig. 7f). ER-α-mediated inhibition of p53 transcriptional activity was suggested to be related to NCOR1 recruitment^49^. We found significant NCOR1 recruitment in the presence of β-E2, upon UVB exposure (Fig. 6g, Extended Data Fig. 7g,h). This was not due to a change in NCOR1 levels (Extended Data Fig. 7h), suggesting that β-E2 enhances the recruitment of the repressor NCOR1 to the ghrelin promotor upon UVB exposure.

To further investigate the mechanism of sex-dependent response to UVB exposure, we blocked estrogen activity in vivo by developing an ovariectomized (OVX) female mouse model (Fig. 6h) which had lower circulating estrogen levels than sham-control mice (Extended Data Fig. 7i). Body weight before and after the OVX surgery significantly increased, as expected^50^ (Extended Data Fig. 6j). Weekly detection of mice food intake in PhenoTypers revealed a significantly higher food intake in OVX mice after UVB exposure (Fig. 6i). Furthermore, staircase, open-field and elevated-plus maze behavioral data clearly demonstrate increased feeding behavior and food intake after UVB radiation in OVX mice compared to OVX-control, sham-control and sham-UVB animals (Fig. 6j,k and Extended Data Fig. 7k). Skin ghrelin mRNA and protein levels, as well as circulating ghrelin, were significantly increased upon UVB exposure in OVX mice compared to skin samples of OVX-control, sham-control and sham-UVB animals (Fig. 6l,m, Extended Data Fig. 7l). Skin p53 mRNA level was significantly elevated upon UVB exposure of both OVX and OVX-control mice (Extended Data Fig. 7m). No significant change in ER-α mRNA levels was observed under all tested conditions (Extended Data Fig. 7n). Overall, the OVX mouse data demonstrate that elevation in skin ghrelin levels after UVB exposure by p53 requires the absence of estrogen.

To examine the effect of estrogen blocking in human skin, we used ex vivo adipose tissue of skin explants. First, we validated that estrogen signaling and its cognate receptor ER-α are significantly higher in female human skin than in male human skin (Fig. 6a, Extended Data Fig. 7a). Next, we found that estrogen signaling inhibitor, letrozole, significantly inhibits the expression of aromatase mRNA (Extended Data Fig. 7o) and that ghrelin mRNA expression was significantly induced in UVB-letrozole-treated adipose tissue compared to vehicle or mock-UVB controls (Fig. 6n). Similar results were obtained when the same experiment was performed with human female adipocytes in culture with a UVB dose of 50 mJ/cm^2^ (Fig. 6o). This data show that ghrelin elevation in the skin post UVB exposure is blocked in the presence of estrogen.

Taken together, our findings indicate that p53 induces ghrelin expression in skin adipocytes in a sex-specific manner and estrogen blocks p53 activity, impeding ghrelin expression in females post-UVB exposure.

### Solar radiation induces ghrelin and hunger in humans

**Fig. 7:**
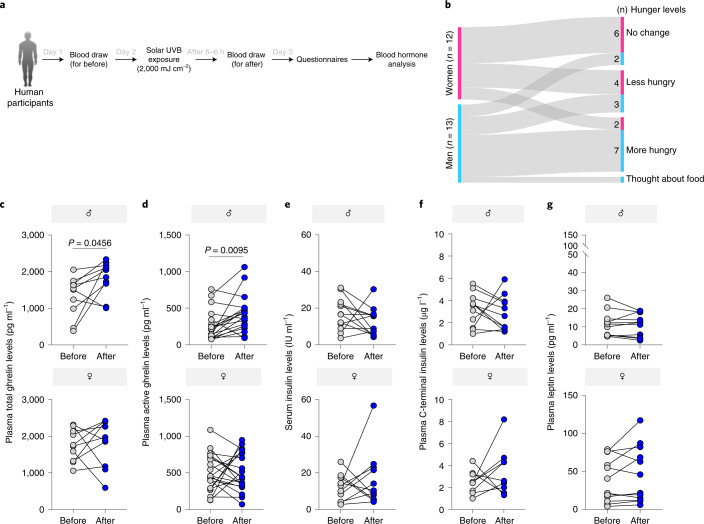
Solar exposure induces ghrelin and hunger in humans. **a**, Experimental design. **b**, Sankey diagram of the number of women (pink) and men (cyan blue) with indicated responses to questions related to thoughts about food and hunger levels (*n* = 13 biologically independent human male subjects and *n* = 12 biologically independent human female subjects). **c**–**g**, Blood plasma levels of indicated hormones on the day before and 5–6 h after a single solar UVB (2,000 mJ/cm^2^) exposure. (**c**) Blood plasma levels of total ghrelin (*n* = 10 biologically independent human subjects per sex). (**d**) Blood plasma levels of active ghrelin (*n* = 18 biologically independent human male subjects and *n* = 20 biologically independent human female subjects). (**e**) Blood serum levels of insulin (*n* = 12 biologically independent human male subjects and *n* = 11 biologically independent human female subjects). (**f**) Blood plasma levels of C-terminal insulin (*n* = 11 biologically independent human male subjects and *n* = 9 biologically independent human female subjects). (**g**) Blood plasma levels of leptin (*n* = 11 biologically independent human male subjects and *n* = 12 biologically independent human female subjects). In all relevant panels: Data are presented as mean ± SEM; the changes before and after for each subject; a paired two-tailed *t*-test *p*-values are shown; statistical details for sex or UVB factors in the ANOVAs (*F*-values, degrees of freedom, *p*-value) with interaction appears in Supplementary Table 10, or two-way ANOVA analysis with multiple correction test appears in Supplementary Table 11. Source data

**Extended Data Fig. 8:**
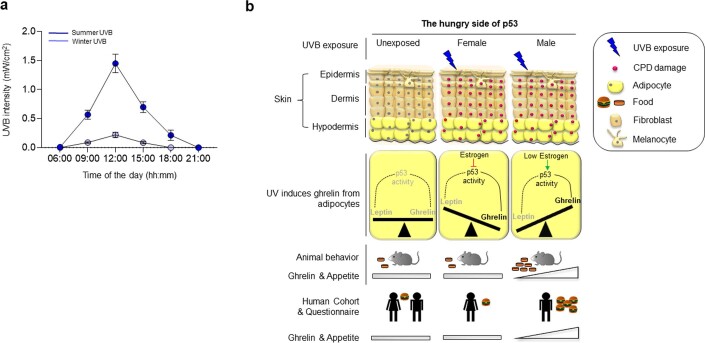
Solar exposure induces ghrelin and hunger in humans, Related to Fig. 7. **a**, Dot plot representing the solar UVB radiation dose (measured in mW/cm^2^), measured using the UVX radiometer at indicated times of the day in summer (August) and winter (December) months. Data are presented as mean ± SEM for each time point of the day (*n* = 4 separate days of measurement). **b**, Graphical summary presents the discovery of UVB as a novel food-seeking behavior trigger, in which men and women have a different eating physiology and in which several extrinsic as well as intrinsic factors can affect this behavior, including sex-specific steroids like estrogen and testosterone. The sex-based disparity in ghrelin levels following the UVB exposure are reflected in the food-seeking behavior and hunger, as we observed in several experimental models, that is, mouse experiments, human cohort study and human questionnaire.

To further understand how solar radiation exposure alters appetite in humans, we asked volunteers (*n* = 13 men and *n* = 14 women; age 18–55 years) to spend about 25 min of exposure to the sunlight (equivalent to 2,000 mJ/cm^2^ UVB) (Extended Data Fig. 8a). Blood samples were collected between 17:00–18:00 hours on the day before and the day after the solar exposure. After the exposure, subjects were asked questions regarding their appetite by a professional psychotherapist (Fig. 7a). We found that men felt significantly hungrier compared to their regular hunger level, whereas women reported no significant difference in their hunger levels (Fig. 7b). These results are in line with the behavioral data obtained in mice and with reports from human subjects treated with phototherapy.

Further, total ghrelin levels were significantly increased in UVB-exposed men compared to the levels the day prior to the experiment (Fig. 7c). Prior to the UVB exposure, total ghrelin levels were higher in women than in men, which was expected^51^, but there was no change in total ghrelin levels in the women after the solar exposure (Fig. 7c). We further quantified the active form of ghrelin (acyl ghrelin) in the plasma and found a significant elevation in solar-exposed men, whereas solar exposure did not alter ghrelin levels in women (Fig. 7d). Further, since reduction in insulin can also explain increase in appetite^52^, we measured serum insulin and C-terminal peptide levels. No significant differences were observed in the blood insulin and C-terminal peptide levels following the UVB treatment (Fig. 7e-f). No significant difference was detected in the circulating leptin levels following solar exposure in either men or women (Fig. 7g). These data suggest that solar exposure equivalent to a dose of 2,000 mJ/cm^2^ UVB enhances appetite and blood ghrelin (total and active forms) plasma levels only in men.

## Discussion

Our study revealed that UVB exposure enhances food-seeking behavior in males via ghrelin and a process that is prevented in females. We found that p53 mediates transcription of *ghrelin* in skin adipocytes and that estrogen interferes with the p53-mediated transcriptional activity, thus blocking the positive effect of solar exposure on food-seeking behavior in females. Previous studies depicted seasonal influences on plasma levels of ghrelin with the highest peak in the summer^53^, in accordance with our findings, however, no direct mechanistic link was suggested. UVB-induced change might be dependent on the amount of skin exposed. In our study, dorsal mouse body was shaved and exposed area accounts for approximately 50–60% of total body area. For the human cohort study, subjects wore sleeveless shirts and shorts, leaving most of the body unexposed. It is highly reasonable that the UVB effect is dependent on the amount of skin exposed.

The increased hypothalamic expression of the orexigenic pathway indicates that ghrelin activated this pathway, which translated to increased food intake in males. As ghrelin serves as a peripheral satiety signal, its concentrations change in response to environmental cues and reach hypothalamic satiety centers. Further, both the opioid and ghrelin axes are modified by UVB exposure and both effect appetite^54^. However, our results indicate that these two pathways are somewhat separate, since the use of opioid antagonists (naltrexone) only partially represses food uptake upon UVB exposure. Ghrelin is not the only peripheral signal that influences hypothalamic satiety centers and it would be interesting to further explore the additional regulators that are activated upon specific environmental cues.

We showed that the male-specific effects of UVB on food-seeking behavior are p53 dependent. p53 is considered the “guardian of the genome” due to its critical role in the cell’s response to DNA damage^35^. Ghrelin enhances p53-dependent DNA repair, for example, in the presence of chemotherapeutic agents such as cisplatin^55^. Leptin, which produces the opposite effect of ghrelin, decreases p53 levels by stimulating Protein kinase C translocation to the plasma membrane and by enhancing ERK1/2 activity in adipocytes^56^. These pieces of evidence, together with our work, implicate p53 as a central mediator of food intake. It is possible that additional stresses that induce p53 activity, such as γ-radiation, oxidative stress, hypoxia and infrared radiation^57–59^, may also regulate appetite through effects on p53.

Upon ionizing radiation, ER-α binds directly to p53^46^ to promoters of its target genes^49^. p53 transcriptional activity and dynamics are fundamentally different after ionizing radiation treatment than after UVB treatment^60^, leading to different cell fate. Cells that experience p53 pulses after ionizing radiation recover from DNA damage, whereas cells exposed to sustained p53 expression after UVB treatment frequently undergo senescence^60^. It is therefore expected that ionizing radiation and UVB will activate different p53 regulatory mechanisms. On the organism level, DNA damage increases plasma levels of β-endorphin^7^ and, according to our findings, UVB boosts plasma ghrelin levels. It will be intriguing to examine whether other endocrine system-related molecules that are induced upon DNA damage impact appetite or upon other skin triggers (for example, heat, touch, etc.).

A direct link between the skin and the brain was demonstrated in a study showing that UVB exposure induces the release of urocanic acid from the skin to the blood, which is converted to glutamate in brain neurons, leading to improvements in motor learning and object recognition in mice^61^. Ghrelin enhances learning and has anti-anxiety effects and neuroprotective functions^32^. It will be interesting to further study whether skin-mediated induction of ghrelin production directly improves brain function, such as memory and learning abilities and whether there are additional hormones released from the skin that modify human behavior.

UV is a well-established carcinogen, but avoiding the sun rays adversely impacts human health, too^11^. Since ghrelin has anti-inflammatory properties^62^, halts heart muscle wasting^63^ and decreases arterial pressure^64^, ghrelin may be the mechanistic link between solar exposure and cardiovascular disease reduction^11^. Moreover, ghrelin enhances insulin sensitivity in metabolic syndrome patients^65^ and in animal models of type II diabetes^66^. Patients who suffer from appetite loss, which affects their health and recovery rate, such as patients undergoing chemotherapy^67^ patients, should benefit from treatments that induce ghrelin production. Indeed, ghrelin administration during chemotherapy has been shown to increase food intake and appetite^68^. The ghrelin receptor is expressed in the brain^69^ and in peripheral tissues (that is spleen, myocardium, thyroid, pancreas, and adrenals)^70^. In addition to its function in regulating energy homeostasis^32^ and the functions mentioned above, ghrelin also mediates glucose homeostasis^71^, muscular atrophy^72^, bone metabolism^73^, stress and anxiety^74^, adipogenesis^75^ and the immune system^76^. Therefore, the various role of ghrelin, might be also observed upon UVB/solar exposure and will be interesting to investigate. Thus, our study suggest that the use of phototherapy might be extended.

## Methods

### Mouse models

All animal experiments were performed in accordance with the guidelines of the Tel Aviv University Institutional Animal Care and Use Committee with institutional policies and approved protocols (IACUC permit: 01-15-086 and 01-19-003). All mice were housed in individually ventilated cages (IVC) (Maximum 5 mice per cage unless mentioned in the experiments) for 12 h dark/12 h light phases with 22 ± 1 °C temperature and 32–35% humidity with *ad libitum* water and food unless mentioned the experiments. Wild-type C57BL/6 mice aged 6–8 (male or female) weeks were purchased from Envigo (Code No #057).

#### p53 conditional knock-out in adipocytes

p53^flx/flx^ mice^77^ were a gift from Professor Eli Pikarksky (The Hebrew University of Jerusalem, Israel) and mice with the *Fabp4* promoter directing expression of *Cre* recombinase^78^ (Fabp4^Cre+^) (Stock: 005069) were purchased from Jackson Laboratory.

#### Ovariectomized (OVX) mouse model

C57BL/6 female mice (6 weeks old) were anesthetized by i.p. injection (20 mg/kg Xylazine and 200 mg/kg Ketamine in sterile PBS without Ca^2+^ and Mg^2+^) and dorsally shaved. The lubricating ophthalmic ointment was applied to the eyes (Lacrilube®) and Rimadyl (5 mg/kg) administered subcutaneously for postoperative pain relief. A 2–3 cm midline incision was made, and the skin was bluntly dissected from the underlying fascia to reach the abdominal cavity, adipose tissue surrounding the ovary was ligated and the ovary exteriorize. The wounds of the peritoneum were closed using an absorbable monofilament suture (Ethicon, USA) and the animals were injected with Rimadyl (5 mg/kg) (Norbrook Laboratories, UK) and enrofloxacin (Bayer, Germany) subcutaneously and placed in recovery cages (25–27 °C). The animals were housed separately for one week postoperatively for the recovery and were monitored for the development of infection. The experiments were performed 4 weeks post OVX surgery.

### Human skin

Skin explants were obtained from healthy adults (age 22–54 years) undergoing abdominoplasty surgery at the Wolfson Medical Center, Tel Aviv, Israel (Helsinki number: 0015-16-WOMC). Human skin (male and female) was washed in antiseptic (Octenisept, Schülke & Mayr GmbH), resected into 2.0 ×2.0 cm pieces and grown on a keratinocyte-based serum-free (PromoCell) semi-solid medium (0.33% agarose) absorbed in 10% fetal bovine serum (FBS) Dulbecco’s minimal essential medium (DMEM) for 5 days^30^. Every 48 h the skin was transferred to fresh semi-solid medium. Human skin was exposed to UVB exposure or mock-UVB irradiation. To block estrogen signaling, we dissected adipose tissue from human female skin and incubated either with vehicle (DMSO) or 5 µM letrozole for 2 h, followed by control or UVB (500 mJ/cm^2^) treatment and snap-freezing after 24 h.

### Cell culture

3T3-L1, HeLa cells were obtained from ATCC, cultured in DMEM with 10% FBS and 1% penicillin/streptomycin/L-glutamine (Biological Industries). 3T3-L1 cells were differentiated (12–14 days) into mature adipocytes^79^. LiSa-2 cells were a gift from Peter Moeller (University of Ulm, Germany), were cultured in DMEM/F12 (1:1) with HEPES, 10% FBS and 1% penicillin/streptomycin (Biological Industries) and differentiated (7 days) into adipocyte-like cells^80^. H1299 cells with homozygous partial deletion of *p53* and deficient in p53 protein expression were obtained from ATCC and maintained in RPMI 1640 medium supplemented with 10% FBS and 1% penicillin/streptomycin (Biological Industries). Primary human white subcutaneous pre-adipocytes (HWP; PromoCell, female donor) (Cat# C12730; Lot # 419Z023) were cultured in pre-adipocyte growth medium (PromoCell). At 80–90% confluence, differentiation was induced by differentiation medium (PromoCell) for 3 days, followed by culturing in a nutrition medium (PromoCell) that was renewed every 2 days for 6–8 days.

### Human subjects

#### UVB phototherapy questionnaire

A quantitative longitudinal study of 32 patients undergoing treatment for various phototherapy-responsive dermatoses including psoriasis, atopic dermatitis, mycosis fungoides and general pruritus (aged 20–82) was conducted in the Tel Aviv Sourasky Medical Center and Assuta Hospital in Israel (Helsinki 0151-17-TLV and 17-ASMC-17). Skin tone directly affects the amount of UVB that penetrates the skin^37^ and probably influences the response. To avoid this bias, most of the patients in our study had Fitzpatrick Skin Type II-III and their treatment protocol was determined by the physician accordingly (that is, higher skin tone will receive higher dose). All the participants were recruited by convenience sampling and asked to sign an informed consent form. The sample consisted of 43.7% males and 56.3% females. Data were collected through self-reported questionnaires^29^ (Hebrew) before exposure to the UVB dose (T1) of 0.1–2.5 J/cm^2^ for 10–12 exposure sessions for a month and after the treatment (T2). Although the skin tone determined the dose starting point, the starting point of the UV dose is less meaningful than total dose, since a continuous treatment with an increasing dose at every additional exposure, was used resulting in a total dose that was the same for all patients regardless of their skin tone. The data were integrated and analysis demonstrated a similar trend for all patients, strongly suggesting no “batch-effect”. The Helsinki approval included two dermatologists, Dr. Mor Pavlovsky and Dr. Hagit Matz, each patient was evaluated by the same dermatologist before and during the phototherapy session.

#### Human cohort study

Subjects were drawn from researchers (aged 18–55) at the Sackler Faculty of Medicine (Tel Aviv University, Israel). To avoid skin tone bias^37^, all the participants in our study had Fitzpatrick Skin Type II-III. Tel Aviv University Ethics Committee approved the study (#0000668-2) and all participants were recruited by convenience sampling and asked to sign an informed consent form. The sample consisted of 48.14% males and 51.85% females. The solar UVB exposure dose was measured using the UVX radiometer (Ultra-Violet Products) at three random places in Tel Aviv University in summer between 11:00–13:00 hours, Israel standard time (IST). The solar UVB exposure time equivalent to the UVB dose of (2,000 mJ/cm^2^) was around 25–30 min of direct sun exposure. The blood draw was done by a certified physician, Dr. Tom Ben-Dov and nurse Yael Bornstein from Tel Aviv University, Israel. The first blood sample was drawn (intravenous; 10 CC) on the day before solar exposure (between 16:00–18:00 hours, IST). The next day, subjects were exposed to the solar UVB radiation (2,000 mJ/cm^2^) between 11:00 to 13:00 hours, IST and second blood sample was drawn (intravenous; 10 CC) 5–6 h post-solar UVB exposure (between 16:00–18:00 hours, IST). Plasma and serum were separated from the blood and stored at −80 °C until further processing. All the subjects were requested to minimize their solar exposure during the 2 days before the experimental day. On the day of sun exposure, subjects were asked to wear shorts and sleeveless clothes and to have their normal lunch before 14:00 hours. Psychological analysis of the subjects was performed by a certified psychotherapist, Dr. Daphna Liber, a day after the second blood draw. Hunger analysis data is represented in Supplementary Table 7. As a compensation, the human subjects were given coffee and pastry vouchers each time they underwent the blood draw session.

#### Human dietary intake

The mean energy consumption for each month in men and women were obtained from the Israeli Ministry of Health’s National Health and Nutrition Survey between 1999–2001. This survey, based on the USDA (United States Department of Agriculture) guide, was conducted by random samples of men and women (age 25–64 years). The MABAT survey received all ethical approvals before conducting the study and all participants provided their consent^81^. A 24-h recall dietary questionnaire was recorded from a random sample of the population registry by trained professionals to quantify total energy consumption. Total energy consumption as well as intake of macro- and micro-nutrients was calculated^82^. Potential seasonal variability was considered by procuring samples of food items during early summer (May) and early winter (December). Samples were purchased at seven different retail supermarkets and grocery stores, ensuring that each individual sample came from a different production lot. Food items were tested for volume and weight between seasons as appropriate for selected products. Energy intake data, specifically the appetite stimulating nutrients (sodium^83^, omega-3^84^, zinc^85^ and iron^86^) and appetite reducing nutrients (carbohydrates^87^, fat, short-chain fatty acids (SCFAs)^88^ and fiber^89,90^) for the men and women are shown in the Supplementary Table 6.

### Statistics and reproducibility

All data are shown as means and standard errors of the mean except for the human energy intake data which is shown as standard deviation of the mean. We used a random experimental design, Student’s *t*-tests (two-tailed) for two-group comparisons and ANOVAs for multiple group comparisons (followed by either Bonferroni’s, Tukey’s or Šídák’s multi-comparison tests), or their non-parametric equivalents. Two-way ANOVA (for indicated groups) analysis with the indicated multiple correction test was performed appears in Supplementary Table 11. For the analysis of the human dietary data; to check the homogeneity of the variances, *F*-test was performed followed by unpaired *t*-test assuming unequal variance with Welch’s correction. Generalized linear model adjusted for age was used to determine the seasonality effect between genders, including the interaction (sex X season) and months along the year. Additional statistical models for analysis were the interaction model, season analysis age-adjustment model and monthly analysis with variables like sex, season, age, and month of the year appears in Supplementary Table 10. Paired human phototherapy questionnaires data with one-tail were statistically analyzed by Wilcoxon tests to examine within-group differences (ranks of T1 vs. T2 for each sex separately). Wilcoxon tests were used to examine the within-group differences (ranks of T1 vs. T2 for each sex separately). Mann-Whitney tests were performed to examine between-group differences (ranks of male participants vs. female participants at each time-point). For human plasma hormone analysis, two-tailed paired *t*-test was performed. All the softwares used in the study appears in Supplementary Table 12. The number of replicates of the biological samples (mice, human tissues, cells, etc.) used in the particular experiment is mentioned in the figure legends for each experiment. The biological replicates for the Fig. 4a,b (*n* = 3 biologically independent human donors), 4f (*n* = 3 biologically independent mice), 6c (*n* = 2 biological independent experiments) and for Extended Data Fig. 4c-d (*n* = 2 biological independent experiments), 4g–4h (*n* = 3 biologically independent mice), 4j (*n* = 2 biological independent experiments), 6a (*n* = 2 biological independent experiments), 6h (*n* = 2 biological independent experiments) will be available upon request to the authors. Adjustments of the red fluorescence color channel in the microscopy (Ghrelin expression) was necessary on our “merged” images. Enhancement was even through the whole individual figure and thought all fluorescence images, to keep the ability to compare between images.

Additional methods, including references, are available in the supplementary information of this manuscript. All relevant ethical regulation authorities: Tel Aviv University Institutional Animal Care and Use (for mouse studies), Wolfson Medical Center (for human skin), The University Ethics Committee (for UVB phototherapy questionnaire and human cohort study) approved the study protocol.

### Reporting summary

Further information on research design is available in the Nature Research Reporting Summary linked to this article.

## Supplementary information

Supplementary Information Supplementary Methods, Supplementary References.

Reporting Summary

Supplementary Video 1 (Related to Extended Data Fig. 4e). Representative 3D construction of immunofluorescence image of human skin adipose tissue of UVB (2,000 mJ/cm^2^) 24 h post-treatment stained for ghrelin (red), Plin1 (green), and nuclei (DAPI, blue) (scale bars, 5 µm). The image reconstructed with the aid of Imaris 3D software is shown as the representative image.

Supplementary Video 2 (Related to Extended Data Fig. 5f; Left panel). Representative 3D reconstructed image, using Imaris 3D software, construction of immunofluorescence image of human skin adipose tissue male men skin adipose tissue 24 h of after UVB (2,000 mJ/cm^2^); the tissue was stained 24 h post-treatment stained for p53 (red), Plin1 (green), and nuclei (DAPI, blue) (scale bars, 5 µm). The image reconstructed with the aid of Imaris 3D software is shown as the representative image.

Supplementary Video 3 (Related to Extended Data Fig. 5f; Right panel). Representative 3D reconstructed image, using Imaris 3D software, construction of immunofluorescence image of human skin adipose tissue male women skin adipose tissue 24 h of after UVB (2,000 mJ/cm^2^); the tissue was stained 24 h post-treatment stained for p53 (red), Plin1 (green), and nuclei (DAPI, blue) (scale bars, 5 µm). The image reconstructed with the aid of Imaris 3D software is shown as the representative image.

Supplementary Table 1 Human proteome mass spectrometry.

Supplementary Table 2 Mass spectrometry and gene ontology GO analysis for mice.

Supplementary Table 3 Predicted upstream transcription regulators of UVB effect.

Supplementary Table 4 Predicted transcription factor binding to Ghrelin promoter (PROMO analysis).

Supplementary Table 5 Predicted upstream regulators of UVB effect.

Supplementary Table 6 Human energy intake data.

Supplementary Table 7 Human cohort psychological hunger analysis.

Supplementary Table 8 Direct solar radiation data.

Supplementary Table 9 Oligonucleotides used in this study.

Supplementary Table 10 ANOVA statistics.

Supplementary Table 11 Two-way ANOVA with multiple correction test used in the study.

Supplementary Table 12 Source Table.

Supplementary Table 13 Antibodies list.

## Figures and Tables

**Extended Data Fig. 9:**
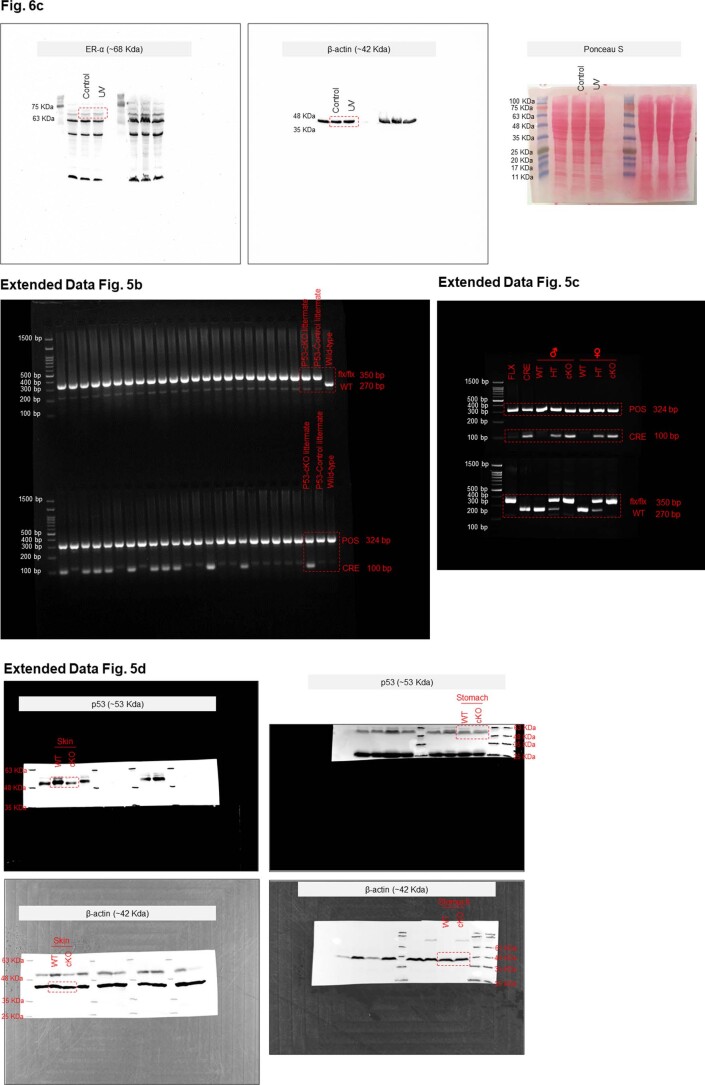
Unprocessed scans of western blots & DNA agarose gel electrophoresis. Unprocessed scans from the western blot experiments from Fig. 6c, and Extended Data Fig. 5d. Unprocessed scans of the genotyping experiments from Extended Data Fig. 5b–c).

**Extended Data Fig. 10:**
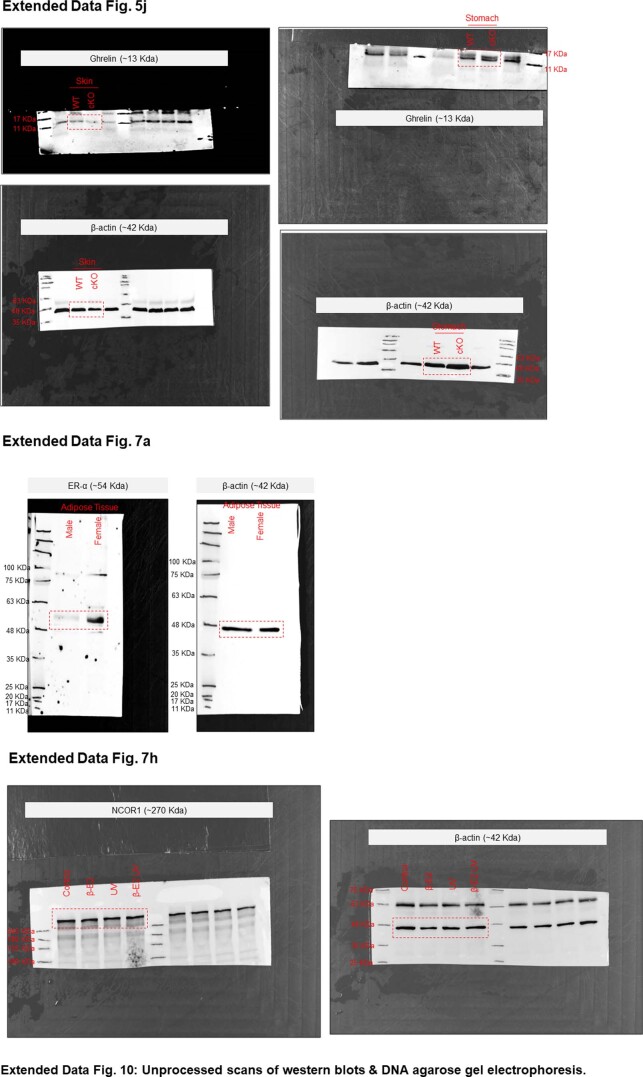
Unprocessed scans of western blots. Unprocessed scans from the western blot experiments from Extended Data Figs. 5j, 7a, and h.

## Data Availability

All original datasets have been deposited at the ProteomeXchange Consortium via the PRIDE partner repository and is publicly available as of the date of publication: Database: PXD033203. Source data provided with this manuscript appears as Source Data 1–7 and Extended Source Data 1–7. Detailed information of the statistical analysis for ANOVA (interaction models and variables with *F*-value, degrees of freedom, actual *p*-value) used in the study appears in Supplementary Table 10. Detailed information of type of ANOVA, multiple correction test used and the *p*-value for all relevant figures appears in Supplementary Table 11. Detailed information about the resources used in the study appears in Supplementary Table 12. All other data can be made available from the authors on reasonable request. The biological replicates for the Fig. 4a,b (*n* = 3 biologically independent human donors), 4f (*n* = 3 biologically independent mice), 6c (*n* = 2 biological independent experiments) and for Extended Data Fig. 4c-d (*n* = 2 biological independent experiments), 4g–4h (*n* = 3 biologically independent mice), 4j (*n* = 2 biological independent experiments), 6a (*n* = 2 biological independent experiments), 6h (*n* = 2 biological independent experiments) will be available upon request to the authors. Source data are provided with this paper.

## Source data

Source Data Fig. 1 Statistical Source Data.
Source Data Fig. 2 Statistical Source Data.
Source Data Fig. 3 Statistical Source Data.
Source Data Fig. 4 Statistical Source Data.
Source Data Fig. 5 Statistical Source Data.
Source Data Fig. 6 Statistical Source Data.
Source Data Fig. 7 Statistical Source Data.
Source Data Extended Data Fig. 1 Statistical Source Data.
Source Data Extended Data Fig. 2 Statistical Source Data.
Source Data Extended Data Fig. 3 Statistical Source Data.
Source Data Extended Data Fig. 4 Statistical Source Data.
Source Data Extended Data Fig. 5 Statistical Source Data.
Source Data Extended Data Fig. 6 Statistical Source Data.
Source Data Extended Data Fig. 7 Statistical Source Data.
