# Supplementary material for: Food-seeking behavior is triggered by skin ultraviolet exposure in males
Source: Nat Metab. 2022 Jul 11;4(7):883–900. doi: 10.1038/s42255-022-00587-9 (PMC9314261; doi:10.1038/s42255-022-00587-9)
Supplement: Supplementary file 1 — Supplementary Methods, Supplementary References. [file 42255_2022_587_MOESM1_ESM.pdf]

nature portfolio

https://doi.org/10.1038/s42255-022-00587-9

Supplementary information

Food-seeking behavior is triggered by skin ultraviolet exposure in males

In the format provided by the authors and unedited

Supplementary information

Methods

Reagents

To mimic the *in-vivo* conditions, cells were stimulated with 5α-dihydrotestosterone (DHT, Sigma-Aldrich) or estradiol (β-E2, Sigma-Aldrich) a day before transfection or irradiation at final concentrations of 100nM. Naltrexone (Sigma-Aldrich) was injected intraperitoneally (i.p.) with dosing of 5 mg/kg of the body weight 30 minutes prior to the start of the experiment. A second injection was given at least 48 hours after the first to avoid carry-forward effects. Indomethacin, insulin solution (from bovine pancreas), biotin, transferrin, calcium pantothenate, cortisol, and triiodothyronine were purchased from Sigma Aldrich. GO-CoA-Tat (Peptides International) was used at the dose of 192 µg/Kg for mouse experiments and 6µM for cell culture experiments. [D-Lys^3]-GHRP-6 (Tocris Bioscience) was used at 200 nmol/mouse. GO-CoA-Tat and [D-Lys^3]-GHRP-6 were injected into mice i.p., and the animal experiments were performed 1-2 hours post injection. Letrozole (Selleckchem) was diluted in DMSO, as per the manufacturer's instructions. For human skin, 5µM letrozole was used, and for the *in-vitro* cell culture experiments, 100nM was used. The stocks of all the chemicals and reagents were prepared and stored as per the manufacturer's instructions.

UVB irradiation

UVB irradiation

Human and mouse skin cells were exposed to the desired UVB doses in an XX-15 stand equipped with 15-W, 302-nm UVB bulbs (Ultra-Violet Products). UV emission was measured with a UVX radiometer (Ultra-Violet Products) equipped with a UVB-measuring head. The delivered doses were calibrated for UVB emittance. Mice were dorsally shaved (~50-60% of the dorsal region) and were re-shaved every week, as necessary. Mice were exposed to UVB in a chamber designed to allow free movement without climbing on each other[30] during irradiation. Between irradiation of each sex, the chamber was cleaned with Virusolve®+ (Amity International).

Solar UVB irradiation measurement

Solar UVB irradiation dose was measured using a UVX radiometer (Analytik Jena) at three random places in Tel Aviv University and the Weizmann Institute, at 06:00, 09:00, 12:00, 15:00, 18:00 and 21:00 hours. The solar UVB was measured in the months of August 2021 (summer) and December 2021 (winter).

Plasmids

The reporter plasmid containing the human *ghrelin* promoter, pCMV-Neo-Bam human p53 vector, and pcDNA3.1-ER-α were gifts from Professor Masayasu Kojima (Kurume University, Japan), Professor Moshe Oren (Weizmann institute of Science, Israel), and Professor Jason Caroll (University of Cambridge, London), respectively.

Genotyping

DNA was extracted from tail sample in extraction buffer (25mM NaOH, 0.2mM disodium salt EDTA, pH 12) for 60 minutes and neutralized (40mM Tris HCl, pH 5). PCR reactions were performed with 20µl containing 10µl 2X GoTaq® green master mix (Promega), and 0.5µM each of Cre (with positive control) and floxed primers (IDT). Primer sequences are listed in Supplementary Table 9. PCR amplification was performed in a Biometra PCR cycler, with cycling conditions of: 95°C/3 minutes, 35 cycles of 95°C/30 sec, 55°C/1 minute, 72°C/1 minute, and 72°C/5 minutes. The identity of each PCR product was revealed by its size in an agarose gel (Cre-recombinase 100 bp, internal positive control 324 bp, and floxed 390 bp).

Oil Red O Staining

Differentiated cells were fixed with 4% paraformaldehyde for 20 minutes at RT, washed with 60% isopropanol for 5 minutes and incubated with Oil Red O solution (Sigma-Aldrich) for 20 minutes followed by counter-staining with Hematoxylin (HHS16, Sigma-Aldrich) for 45 seconds and mounted with aqueous mounting media (10% gelatin in 50 ml double distilled water and 50 ml glycerol; pH 7).

Blood draws, serum and plasma separation

Mice

Mice were anesthetized using i.p. injection of ketamine (Bremer Pharma GMBH) and xylazine (Eurovet Animal Health). The heart was punctured with 22-G needle (KDL), and blood was collected in EDTA microvette tubes (BD

Microtainer) and maintained on ice until centrifugation at 2000 g for 10 minutes at 4°C. To quantify ghrelin in its acylated, active form, we collected the blood and acidified the plasma as suggested in a previous report[91]. Blood samples were collected in pre-chilled EDTA-coated Eppendorf tubes and centrifuged at 2000g for 15 minutes at 4 °C. The resulting supernatant was collected in the pre-chilled Eppendorf tubes, and to every 50µl of plasma, 2.5 µL of 1 M HCL and 0.5µL of the protease inhibitor phenylmethylsulfonyl fluoride (PMSF, Sigma-Aldrich) to stabilize acyl-ghrelin. Plasma was separated and samples were aliquoted and stored at -80°C until use.

Humans

Blood was drawn from humans cubital fossa (branch of the cephalic vein; either forearms) into Vacutainer® K2E (EDTA) tubes (BD Biosciences) using a blood collecting needle set (KDL). Plasma was separated from the blood as previously described[92]. For analysis of acylated ghrelin, we acidified the blood plasma as suggested previously stored in -80 °C until further use[91].

For serum analyses, venous blood was collected in Vacutainer® clot activator tubes (BD Biosciences)and allowed to clot at room temperature (RT) for 15–30 minutes. The clot was removed by centrifugation at 1,000–2,000g for 10 minutes at 4°C as previously described[92]. Aliquots of serum were stored in -80°C until further processing.

Radioimmunoassays for ghrelin, ACTH, β-endorphin, and α-MSH

Plasma ghrelin, ACTH, β-endorphin, and α-MSH levels after appropriate dilution were detected using the radioimmunoassay kits (Phoenix Pharmaceuticals) as per the manufacturer's protocol.

ELISA for leptin, ghrelin, and acyl-ghrelin quantification

After appropriate dilution the plasma leptin (mouse; Crystal Chem), leptin (human; Bertin), ghrelin (mouse; Cusabio), 17-β estradiol (mouse; Abcam), acyl-ghrelin (mouse or human; Merck Millipore) were detected using an ELISA kit, as per the manufacturer's protocol. Undiluted plasma insulin (mouse; Mercodia) was detected using an ELISA kit, as per the manufacturer's protocol.

Immunoassay for detection of human insulin

Blood serum insulin levels or plasma C-peptide levels were measured by an chemiluminescent immunometric method using an IMMULITE 2000 immunoassay auto analyzer system (Siemens Healthcare Diagnostics, Inc.)

with a solid phase (bead) coated with either murine anti-insulin or anti-C-peptide monoclonal antibodies. The immunoassays were performed as per the manufacturer's instructions.

Immunoassay for detection of human estradiol-17β from skin adipose tissues

Snap-frozen adipose tissue were homogenized in RIPA buffer (0.1% SDS, 50 mM Tris-HCl (pH 8), 1% NP-40, 0.5% deoxycholate 150mM NaCl, 1 mM Na_3VO_4, 5mM PMSF, 17mM β- glycerophosphate, 2.5mM sodium pyrophosphate, 5mM EDTA, and 1:100 protease inhibitors cocktail (Sigma–Aldrich)) and incubated on ice for 1 hour followed by centrifugation at 10,000g for 15 minutes at 4°C. The tissue lysates were analyzed for levels of estradiol-17β (β-E2) measured by solid-phase, enzyme-labeled chemiluminescent competitive immunoassay (Immulite 2000, Siemens Healthcare Diagnostics, Inc.).

Western blotting

Protein preparation from cells

Whole cell protein lysates were prepared using RIPA buffer with inhibitors and incubated at 4°C for 30 minutes followed by centrifugation at 12,000g for 15 minutes at 4°C. The resulting clear phase was stored at -80°C until analysis.

Protein preparation from tissues

Snap-frozen tissues were homogenized in RIPA buffer with inhibitors and incubated on ice for 1 hour followed by centrifugation at 10,000 g for 15 minutes at 4ºC. The resulting clear phase was stored at -80°C.

SDS-PAGE and blotting

Equal amounts of proteins as determined by Bradford quantification were separated on either 10% or 16% SDS-PAGE and then electro-blotted onto a Protran nitrocellulose transfer membrane (GE Healthcare)followed by staining with anti ghrelin (Bioss), anti-ER-α (Cell Signaling Technology), anti-NCOR1 (Cell Signaling Technology), anti-p53 (Abcam), and anti-β-actin (Cell Signaling Technology) primary antibodies for 12 hours at 4°C. HRP-conjugated secondary antibodies: rabbit anti-mouse (Abcam) or goat anti-rabbit (Abcam and Sigma-Aldrich) were used. Antibody information (catalog number, dilution and clones) appears in Supplementary Table 13.

RNA purification and qRT-PCR

RNA from cells and tissues

Total RNA was purified from cultured cells using Trizol™ (Invitrogen) according to manufacturer's instructions. Flash frozen tissues were homogenized with magnetic beads (Next Advance) using a bullet blender (Invitrogen).

RNA from formalin-fixed, paraffin-embedded (FFPE) tissues

Formalin-fixed, paraffin-embedded mice tissues were cut into 30 µm sections followed by de-paraffinization with 100% xylene for 3 minutes at 50°C and centrifuged for 1 minute at 14,000g. The pellet was washed twice with absolute ethanol and air dried. Proteins were digested in 150 µl 1X protease K digestion buffer (20 mM Tris-HCl pH 8 ,1 mM $CaCl_2$, 0.5% sodium dodecyl sulfate and 500 µg/ml proteinase K (New England Biolabs)) at 55°C for 3 hours. RNA was isolated using the TRIzol™ protocol according to the manufacturer's instructions and stored at -80°C until further processing.

RNA from optimal cutting temperature compound (OCT) embedded tissues

OCT embedded sections were thawed and the whole brain tissue were isolated. The hypothalamus was carefully excised and smashed followed by RNA isolation by TRIzol™ protocol and one-step qRT-PCR analyses. For qRT-PCR analyses, 1 µg RNA for cells or 0.5 µg RNA for tissues was subjected to qScript™ cDNA Synthesis Kit (Quanta Bioscience) and PerfeCTa® SYBR® Green FastMix® (Quanta Bioscience).

One-step qRT-PCR

For one-step qRT-PCR analyses, the reaction mixture included MultiScribe™ Reverse Transcriptase enzyme (Invitrogen) along with PerfeCTa® SYBR® Green FastMix® and forward/reverse primer mix the with an additional step of cDNA synthesis before the qRT-PCR. Relative expression was normalized either to *18S* or *36b4*. All reactions were performed in duplicates. All primer sequences appear in Supplementary Table 9.

Transfection and luciferase reporter assay

The plasmid constructs (reporter and over-expression together with pCMV-Renilla plasmid) were co-transfected in 12-well plates (2 µg DNA/well) using JetPEI® (Polyplus Transfection) according to the manufacturer's instructions. 24 hours post-transfectioncells were lysed and assayed using dual-luciferase reagents (Promega) as per the manufacturer's instruction. Promoter activity

was normalized to the activity of the constitutively expressed *Renilla* luciferase. All reactions were performed in duplicates.

Immunofluorescence analyses

Immunofluorescence of cells

Cells were fixed with 4% paraformaldehyde (Electron Microscopy Sciences) for 20 minutes, stained with ghrelin (Bioss) and/or Plin1 (Abcam) for 1 hour at RT followed by incubation with Alexa Fluor 488 (Invitrogen) or Alexa Fluor 594 (Invitrogen) for 1 hour at RT. DAPI (Sigma Life-Sciences) was used as nuclear staining. Images were obtained at X20, X40 or X100 magnification using fluorescence microscopy (Nikon) and analysis was done using ImageJ software.

Histology

Human and mouse skin biopsies were fixed in 4% paraformaldehyde (Electron Microscopy Sciences). Stainings were performed on 10 µm thickness tissues. For immunofluorescence section were stained using anti-ghrelin (Bioss), anti-Plin1 (Abcam), anti-CPD (Cosmo), anti-p53 (Cell Signaling Technology) antibodies overnight at 4°C followed by Alexa Fluor 488 (Invitrogen), Alexa Fluor 594 (Invitrogen), Alexa Fluor 594 (Invitrogen) or Alexa Fluor 647 (Invitrogen) secondary antibodies for 1 hour at RT. DAPI (Vector laboratories) was used as nuclear staining. Images were obtained at X4, X10, X20, and X40 magnification using fluorescence microscopy (Nikon) and analysis was done using ImageJ software. Antibody information (catalog number, dilution and clones) appears in Supplementary Table 13.

Tissue section were stained using hematoxylin (HHS16, Sigma-Aldrich) and eosin (Sigma-Aldrich) followed by mounting with DPX mountant (Sigma-Aldrich). Images were obtained with Aperio Slide Scanner microscope (Leica Biosystem, USA), at X20 magnification.

Fontana-Masson staining was performed on the tissue sections as per manufacturer's protocol (Abcam) and images obtained at desired magnification using bright-field microscopy (Nikon).

OCT embedding for brain tissues

Mice brain tissues were dissected and fixed with 4% paraformaldehyde (overnight) and cryo-protected sequentially in 15% and 30% sucrose (Sigma Aldrich) at 4°C. and embedded in OCT medium. .

Chromatin immunoprecipitation (ChIP)

ChIP was performed as described previously[93]. The immunoprecipitations were performed with anti-p53 rabbit polyclonal antibody (Cell Signaling Technology), anti-NCOR1 (Cell Signaling Technology), and normal rabbit IgG as control (Abcam). qRT-PCR was then performed on ChIP samples to amplify specific genomic regions. We designed primers that span p53 and p21 binding sites in the ghrelin upstream region, identified using PROMO and considering the p53 consensus binding motif[94,95]. Relative expression was normalized to input samples. All primer sequences are listed in Supplementary Table 9. Antibody information (catalog number, dilution and clones) appears in Supplementary Table 13.

PhenoTyper analysis

PhenoTyper is an automated system measuring animal behavior in a home cage-like setting. Dorsally shaved mice were exposed to UVB (50 mJ/cm^2) for 4 weeks followed by daily tracking food access by the mice using beam break counts and water access using lickometer. Total activity in the arena and cumulative duration of time spent in the nesting area was tracked for 23 hours (bifurcated into active phase: 19:00-08:00 hours and resting phase: 09:00-19:00 hours). For OVX mice, weekly food intake was monitored manually before and after 24 hours of the PhenoTyper analysis, using electronic scale (MRC lab). The recording of the events was done using Ethovision-XT software (Noldus Information Technology). Mice were counter-balanced, bedding was changed and the PhenoTyper were cleaned once in a week.

Food intake analysis

The food intake for each mice was manually calculated by housing one animal per cage with *ad libitum* access to food and water which allowed the precise measurement of a single animal and minimized the noise during habituation to a new environment, which can affect food intake[25]. The food intake was measured (in grams) at 07:00 and 19:00 hours every day for a duration of 10 weeks (active phase: 19:00–07:00 hour and resting phase: 07:00–19:00 hour), using an electronic weighing scale (MRC Lab). The weekly food intake was averaged for each animal.

Mouse fecal lipids extraction and analysis

Mouse feces (1,000 mg) were collected. For extraction of lipids, 5 ml of saline (Biological Industries) was added to feces, vortexed vigorously and mixed until it formed slurry-like paste. A 5-ml aliquot of chloroform:methanol (2:1) was added to the tubes and vortexed followed by centrifugation at 1,000 g for 10 minutes at RT. The lower liquid phase with the extracted lipids was removed by inserting a 22-G needle through the tube wall and collected in glass tube. The tubes were placed in the fume hood for 3-4 days to evaporate the liquid followed by weighing the tubes on the analytical balance. To calculate the fecal lipids first the weight of the tube was subtracted from the empty tube weight and the fecal lipids were analyzed[96].

Body weight analysis

The bodyweight of the mice (grams) was measured before the start of the experiment and then for indicated times, using an electronic weighing scale (MRC Lab).

Food deprivation regime and habituation

Mice were habituated for 1 week prior to the start of the experiment. All the animals were introduced gradually to a food restriction regime over 7 days: initially with 12 hours feeding, gradually reduced to 8, 5, 3, and 2 hours feeding per day as described by Lopez *et al.* with slight modifications[97]. The 2 hours feeding time was sustained, and animals were prevented from falling below 90% of free-feeding body weight with additional feeding when necessary. From the first food deprivation, the animals were exposed to a drill with the peanut butter-flavored sucrose pellets (14 mg/pellet; Test Diet) placed inside the cage after the starvation period for the motivation. The mice were brought into the experimental rooms (staircase, open-field, hot plate test or elevated-plus maze) 3 days prior to experiment and were kept for 30 minutes to allow habituation to the environment.

Staircase test

For staircase training sessions, mice on food deprivation were fed at noon on non-testing days with water *ad libitum*. At the outset of testing, the mice were first familiarized with the reward food and then to the test boxes by placing food along the surface of the central trough or on the staircase steps for a 10 minutes session a day prior to the experimental day, to rule out the effects of neophobia. On the test day the mice were placed in the staircase chamber one animal at a

time[26]. A mirror was used in order to check the pellets consumed from both the sides. The duration of the test was of 10 minutes, and pellets eaten and attempts were manually scored for each animal. Between trials, the staircase chamber was cleaned with Virusolve®+ (Amity International). The sessions of the test were recorded and scored using media recorder (Noldus Information Technology).

Open-field test

Open-field consisted of 50 × 50 cm square arena with a white floor andlit by normal white light (300 Lux) in the experiment room. The test was initiated by placing a single mouse in a corner of the arena and allowing free movement for 15 minutes. The reward food (~100 food pellets) was kept in the center of the arena in a 50-ml tube cap (green color) glued to the arena. For the activity analysis, the food was excluded from the arena. Mouse behavior (time spent in the center, total distance traveled in the arena, velocity and activity levels) were measured by video tracking of the animal in the arena and by the generation of a heat map by EthoVision. The number of the pellets consumed and defecation were manually assessed. Between each trial, the open-field arena and the tube cap were cleaned with Virusolve®+ (Amity International) to avoid odors contamination. The test session videotapes were scored and analyzed with EthoVision-XT software (Noldus Information Technology).

Elevated-plus maze (EPM) test

The test was performed using EPM[98] apparatus set at a height of 90cm. The maze was lit by normal white light (300 Lux) in the experiment room. The maze included two opposing closed arms (arm lengths 40 cm, arm widths 5 cm, wall height 15 cm), two opposing open arms (lengths 40 cm, widths 5 cm) and an open 5 X 5 cm square in the center. An animal was placed in the center facing an open arm, and behavior-related parameters were measured for 5 minutes using the camera from the top and analyzed using EthoVision-XT software (Noldus Information Technology). Between each trial, the maze was cleaned with Virusolve®+ (Amity International).

Respirometry analysis

VO_2, which is the maximum amount of oxygen the mouse body can consume at physiological level, was measured using a flow through respirometry[99] for dorsally shaved mice exposed to UVB (50 mJ/cm^2) for 4 weeks, and control

mock-UVB treated mice. After gradual starvation, mice were subjected to the metabolic chamber analysis. In each measurement seven post-operative mice were placed in individual chambers (4 L) inside a temperature-control cabinet (Panasonic, Japan) at 29°C (the thermal neutral point for mice during resting phase[100]). Mice received dry CO_2 free air in a flow rate of 400 ml/second (V8, eight-channel flow mass control, Sable Systems). An empty cell was used as a baseline reference. Air from the chambers went through eight channel multiplexer (Sable Systems) to a Li-7000 CO_2/H_2O analyzer (Licor) and through a column of magnesium perchlorate and Ascarite® (water and CO_2 adsorbents, respectively) into an Oxzilla O_2 analyzer (Sable Systems). We let mice to acclimate for 60 minutes and measured each mouse for 15 minutes twice. Baseline of 5 minutes were run every 30 minutes. VO_2 was calculated using equation 10.1[99].

Hot plate test

Mice underwent thermal analgesic testing during UVB/mock treatment regimens using the hot plate test[7]. Naltrexone (5 mg/kg) or saline was i.p. injected 15 minutes prior to the start of the experiment. The temperature of the hot plate (Ugo basile®) was maintained at 52°C and the locomotion was restricted by a Plexiglas cylinder. The latency until the first response (hind paw flutter, hind paw licking or jumping), an indicator of the nociceptive behavior, was recorded, and at this point, the animal was immediately removed from the hot plate and brought back to the home cage.

Proteolysis and mass spectrometry

The frozen aliquots of the blood plasma from mice or humans were sent to the Smoler Proteomics Center at Technion, Israel, for mass spectroscopy analysis.

Mice

The peptides were separated on a Cutoff filtration (3 kDa) on Amicon® Ultra Centrifugal Filters followed by desalting on C18 tips of the filtrate and re-suspending in 0.1% Formic acid from 50 µl plasma and analyzed by LC-MS/MS on Q Exactive plus (Thermo Fisher) and identified by Proteome Discoverer 1.4 by comparison to the mouse Uniprot database. In order to get more information on the small proteins in the samples were trypsinized and analyzed in a similar way. Semi analysis was done by calculating the peak areas for each peptide. The intensities between samples were normalized to the intensities of four of

major serum proteins and transformed to log2 intensities. The area of the protein was taken as the average of the three most intense peptides from the protein. The IgG proteins were deleted for the significance analysis. The raw data with the statistical analysis is shown in Supplementary Table 2. Mice plasma proteomics upon UVB exposure was previously published by us under accession number ProteomeXchange Dataset PXD025973.

Human

The proteins from the different samples were precipitated on 90% Ethanol, at 90°C for 10 minutes, followed by 10,000 g centrifugation for 5 minutes. The supernatant was dried and re-suspended in 9 M Urea, 400 mM Ammonium bicarbonate, reduced with 3 mM dithiothreitol (60°C for 30 minutes), modified with 12 mM iodoacetamide in 400 mM ammonium bicarbonate (in the dark, RT for 30 minutes) and digested in 1 M Urea, 50 mM ammonium bicarbonate with modified trypsin (Promega) at a 1:50 enzyme-to-substrate ratio, at 37°C for 2 hours. The tryptic peptides were desalted using C18 tips (Top tip, Glygen) dried, and re-suspended in 0.1% Formic acid. The peptides were resolved by reverse-phase chromatography on 0.075 X 180-mm fused silica capillaries (J&W Scientific) packed with reproSil reversed-phase material (Dr. Maisch GmbH). The peptides were eluted by way of a linear 60-minute gradient of 5 to 28%, 15-minute- gradient of 28 to 95%, and 25 minutes at 95% acetonitrile with 0.1% formic acid in water at a flow rate of 0.15 µl/minute. Mass spectrometry was performed with an Q Exactive HF mass spectrometer (Thermo Fisher) in a positive mode using repetitively full MS scan followed by collision induced dissociation (HCD) of the 18 most dominant ions selected from the first MS scan. Normalization was done using the 5 most intense proteins: Apolipoprotein A2 (APOA2), Hemoglobin alpha 1 (HBA1), Alpha-2-HS-glycoprotein (AHSG), Beta globin (HBB), and Albumin (ALB). The raw data is shown in Supplementary Table 1.

Direct solar radiation data

Solar radiation is the mixture of different wavelengths of UV radiation[101]. Radiation values where downloaded from the Israeli Meteorological website (www.ims.gov.il). Direct radiation measurements (KJ/m^2) were obtained from 03:00–17:00 (UTC time) of each day between January 1999 and February 2001

for the Haifa region (32.81°N). Daily-monthly average direct solar radiation data is shown in Supplementary Table 8.

Skin pigmentation measurements

Skin pigmentation was measured by reflectance spectrophotometer (Derma spectrophotometer, Cortex Technology) on the ears of the mice after standard calibrations according to the manufacturer's instructions. Three readings were taken at each site per mice. All skin color examinations were made at RT in conditions devoid of sunlight.

UVB phototherapy subject questionnaire

We used the Disease-Related Appetite Questionnaire (DRAQ;[29]), translated into Hebrew for our study. The questionnaire was developed to investigate Nutrition Impact Symptoms, mainly in patients suffering from, or at risk of, developing malnutrition and can be used in different kinds of medical diagnoses, concerning appetite, hunger, and other eating-related issues. In our study, as we focused on changes in appetite and hunger, we omitted items that were irrelevant (e.g., how the food tasted). The questions were rated on a 3-point multiple-choice scale, with higher scores indicative of increased appetite. Due to the small range of the scale (1-3) we used the Wilcoxon test, a non-parametric test, to examine within-group differences (ranks of T1 vs. T2 for each sex separately).

Psychological analysis of human cohort exposed to solar radiation

Participants were asked questions by a certified psychology expert. The questions asked following the UVB exposure addressed whether i) the person felt hungrier, ii) the person felt less hungry, or iii) there was no change in the hunger levels and iv) whether the person thought about the food during the experiment.

3D image reconstruction of p53 and ghrelin in skin adipose tissue

Immunostaining of formalin-fixed, paraffin-embedded human skin tissues was performed as described above. The Z-stack series of images were obtained using a Leica SP5 confocal microscopy at X63 magnification and were reconstructed into 3D animations using Imaris 3D software (version 8.4.1). 3D image construction to show specificity ghrelin and p53 staining in adipose tissue, as shown in Extended Data Figs. 3e and 4f and supplementary Videos 1, 2 and 3).

ImageJ analysis

Ghrelin quantification in human skin tissue

For ghrelin intensity quantification, the X10 or X40 images taken from human skin following treatments were split into separate channels and converted to 8-bit images using ImageJ. Ghrelin intensity was normalized to DAPI from the same image to rule out discrepancies due to differences in cell numbers.

p53 quantification in human skin tissue

For p53 intensity quantification, the X10 images with more than 20 cells per field of the human skin following the indicated treatments were considered for analysis. Fluorescence images were quantified as described above.

Ghrelin quantification in mouse tissue

For ghrelin intensity quantification, the X40 images of skin samples from mouse following the indicated treatments were quantified as described above.

For ghrelin intensity quantification, the X20 images of stomach from mouse with indicated treatments were quantified as described above.

Ghrelin quantification in LiSa-2 and 3T3-L1 adipocytes

For ghrelin intensity quantification, the X40 images of the cells with indicated treatments were quantified as described above.

Ingenuity Pathway Analysis

The raw data from the blood plasma mass spectrometry was subjected to "Upstream Regulator Analysis" for males and females (UVB normalized to mock-UVB treatment) separately. After analysis, *p*-values were computed separately for males and females providing the list of the upstream regulators (transcription factors, chemical, drug, kinase, and other complexes). The list of the upstream predictor transcription factors is shown in the Supplementary Table 3 and the potential predicted up-stream chemicals are shown in Supplementary Table 4.

Promoter analysis

A total of 23 potential p53-binding sites were identified in six regions from the human ghrelin promoter (-3000 base pairs upstream of the transcription start site) was procured from ensemble, and this sequence was analyzed using PROMO 3.0 (version 8.3, ALGGEN Research Software) to identify putative transcription factor binding sites[102].

Mass spectrometry and proteomap analysis

The mass spectrometry human data (before and after a single solar exposure, five biological repeats) were analyzed using MaxQuant software 1.5.2.8 (Mathias Mann group, Max Planck Institute of Biochemistry) vs. the human proteome from the Uniprot database with 1% FDR (false discovery rate). The data were quantified by label-free analysis using the same software. Statistical analysis for identification and quantization results was done using Perseus 1.6.10.43 software. Proteomaps were constructed using a web tool (Bionic Visualizations version 2.0) (proteins >2 fold difference) before and after UVB exposure (higher expression) in males and in females[103]. Each polygon corresponds to a functional category according to KEGG pathway annotations, and the size corresponds to the fold change.

References

91. Azzam, I., Gilad, S., Limor, R., Stern, N. & Greenman, Y. Ghrelin stimulation by hypothalamic–pituitary–adrenal axis activation depends on increasing cortisol levels. *Endocr. Connect.* (2017) doi:10.1530/EC-17-0212.
92. Renshaw, A. Henry??s Clinical Diagnosis and Management by Laboratory Methods. *Adv. Anat. Pathol.* (2007) doi:10.1097/pap.0b013e31803255cc.
93. Glaich, O. *et al.* DNA methylation directs microRNA biogenesis in mammalian cells. *Nat. Commun.* (2019) doi:10.1038/s41467-019-13527-1.
94. Briones-Orta, M. A. *et al.* Prediction of transcription factor bindings sites affected by SNPs located at the osteopontin promoter. *Data Br.* (2017) doi:10.1016/j.dib.2017.07.057.
95. Farré, D. *et al.* Identification of patterns in biological sequences at the ALGGEN server: PROMO and MALGEN. *Nucleic Acids Res.* **31**, 3651–3653 (2003).
96. Kraus, D., Yang, Q. & Kahn, B. Lipid Extraction from Mouse Feces. *BIO-PROTOCOL* (2015) doi:10.21769/bioprotoc.1375.
97. Llano Lopez, L., Hauser, J., Feldon, J., Gargiulo, P. A. & Yee, B. K. Evaluating spatial memory function in mice: A within-subjects comparison between the water maze test and its adaptation to dry land. *Behav. Brain Res.* (2010) doi:10.1016/j.bbr.2010.01.020.
98. Pellow, S., Chopin, P., File, S. E. & Briley, M. Validation of open : closed arm entries in an elevated plus-maze as a measure of anxiety in the rat. *J. Neurosci. Methods* (1985) doi:10.1016/0165-0270(85)90031-7.
99. Lighton, J. R. B. *Measuring Metabolic Rates: A Manual for Scientists*. *Measuring Metabolic Rates: A Manual for Scientists* (2008). doi:10.1093/acprof:oso/9780195310610.001.0001.
100. Škop, V. *et al.* Mouse Thermoregulation: Introducing the Concept of the Thermoneutral Point. *Cell Rep.* (2020) doi:10.1016/j.celrep.2020.03.065.
101. Hölzle, E. & Hönigsmann, H. UV-radiation--sources, wavelength,

environment. *J. Dtsch. Dermatol. Ges.* (2005) doi:10.1111/j.1610-0387.2005.04392.x.

102. Messeguer, X. *et al.* PROMO: detection of known transcription regulatory elements using species-tailored searches. *Bioinformatics* **18**, 333–334 (2002).
103. Liebermeister, W. *et al.* Visual account of protein investment in cellular functions. *Proc. Natl. Acad. Sci. U. S. A.* (2014) doi:10.1073/pnas.1314810111.
