# Supplementary material for: Food-seeking behavior is triggered by skin ultraviolet exposure in males
Source: Nat Metab. 2022 Jul 11;4(7):883–900. doi: 10.1038/s42255-022-00587-9 (PMC9314261; doi:10.1038/s42255-022-00587-9)
Supplement: Supplementary file 2 — Reporting Summary [file 42255_2022_587_MOESM2_ESM.pdf]

nature portfolio

Corresponding author(s): Dr. Carmit Levy

Last updated by author(s): May 4, 2022

Reporting Summary

Nature Portfolio wishes to improve the reproducibility of the work that we publish. This form provides structure for consistency and transparency in reporting. For further information on Nature Portfolio policies, see our Editorial Policies and the Editorial Policy Checklist.

Statistics

For all statistical analyses, confirm that the following items are present in the figure legend, table legend, main text, or Methods section.

n/a	Confirmed	
☐	☒	The exact sample size (*n*) for each experimental group/condition, given as a discrete number and unit of measurement
☐	☒	A statement on whether measurements were taken from distinct samples or whether the same sample was measured repeatedly
☐	☒	The statistical test(s) used AND whether they are one- or two-sided<br>*Only common tests should be described solely by name; describe more complex techniques in the Methods section.*
☒	☐	A description of all covariates tested
☐	☒	A description of any assumptions or corrections, such as tests of normality and adjustment for multiple comparisons
☐	☒	A full description of the statistical parameters including central tendency (e.g. means) or other basic estimates (e.g. regression coefficient) AND variation (e.g. standard deviation) or associated estimates of uncertainty (e.g. confidence intervals)
☐	☒	For null hypothesis testing, the test statistic (e.g. *F*, *t*, *r*) with confidence intervals, effect sizes, degrees of freedom and *P* value noted<br>*Give P values as exact values whenever suitable.*
☒	☐	For Bayesian analysis, information on the choice of priors and Markov chain Monte Carlo settings
☒	☐	For hierarchical and complex designs, identification of the appropriate level for tests and full reporting of outcomes
☒	☐	Estimates of effect sizes (e.g. Cohen's *d*, Pearson's *r*), indicating how they were calculated

Our web collection on statistics for biologists contains articles on many of the points above.

Software and code

Policy information about availability of computer code

Data collection	For data collection Proteome Discoverer 1.4 software for the mass spec.<br>Data with the related animal behavior experiments was collected with EthoVision XT 7 (Noldus Information Technology) and media recorder (Noldus Information Technology).
Data analysis	All analyses were done using PROMO 3.0 (version 8.3), Excel 2016 (Microsoft Corp.), Prism 8 (Graphpad Software), SPSS Statistics version 25.0 (IBM), ImageJ (https://imagej.nih.gov/ij/), and paint.net (https://www.getpaint.net/), IMARIS (version 8.4.1), MaxQuant 1.5.2.8, Perseus 1.6.10.43 software.<br>For human ghrelin promoter analysis: The human ghrelin promoter (-3000 base pairs upstream of the transcription start site) was procured from ensemble (https://www.ensembl.org/), and this sequence was analyzed using PROMO 3.0 (version 8.3, ALGGEN Research Software) to identify putative transcription factor binding sites.<br>For solar radiation: Radiation values where downloaded from the Israeli Meteorological website (www.ims.gov.il). Direct radiation measurements (KJ/m2) were obtained from 03:00–17:00 (UTC time) of each day between January 1999 and February 2001 for the Haifa region (32.81°N).

For manuscripts utilizing custom algorithms or software that are central to the research but not yet described in published literature, software must be made available to editors and reviewers. We strongly encourage code deposition in a community repository (e.g. GitHub). See the Nature Portfolio guidelines for submitting code & software for further information.

Data

Policy information about availability of data

All manuscripts must include a data availability statement. This statement should provide the following information, where applicable:

- Accession codes, unique identifiers, or web links for publicly available datasets
- A description of any restrictions on data availability
- For clinical datasets or third party data, please ensure that the statement adheres to our policy

All original datasets has been deposited at the ProteomeXchange Consortium via the PRIDE partner repository and is publicly available as of the date of publication: Database: PXD033203. Source data provided with this manuscript appears as Source Data 1-7 and Extended Source Data 1-7. Detailed information of the statistical analysis for ANOVA (interaction models and variables with F-value, degrees of freedom, actual p-value) used in the study appears in Supplementary Table 10. Detailed information of type of ANOVA, multiple correction test used, and the p-value for all relevant figures appears in Supplementary Table 11. Detailed information about the resources used in the study appears in Supplementary Table 12. All other data can be made available from the authors on reasonable request. The biological replicates for the Fig. 4a-4b (n = 3 biologically independent human donors), 4f (n = 3 biologically independent mice); 6c (n = 2 biological independent experiments) and for Extended Data Fig. 4c-4d (n = 2 biological independent experiments), 4g-4h (n = 3 biologically independent mice), 4j (n = 2 biological independent experiments); 6a (n = 2 biological independent experiments), 6h (n = 2 biological independent experiments) will be available upon request to the authors.

Field-specific reporting

Please select the one below that is the best fit for your research. If you are not sure, read the appropriate sections before making your selection.

☒ Life sciences ☐ Behavioural & social sciences ☐ Ecological, evolutionary & environmental sciences

For a reference copy of the document with all sections, see nature.com/documents/nr-reporting-summary-flat.pdf

Life sciences study design

All studies must disclose on these points even when the disclosure is negative.

Sample size	Sample size was chosen as acceptable in the field of behavioural experiments, upon consulting with leading experts: Dr. Shamgar, Dr. Weller and Dr. Bikovski. Detailed description of the statistical methods used for the analyse, appears in the paper.
Data exclusions	For mouse studies: All our in vivo animal experiments, we always started with the specific number of animals per group, during the experiment due to technical discrepany in the treatment we had to terminate the study for this particular animal.<br><br>For human: Inclusion/exclusion criteria are stated in method section and for the data analysis of the energy intake and the human clinical questionnaires. For the human cohort study experiments, random samples from the subjects were chosen.
Replication	The experimental findings in animals in vivo and in vitro experiment were reproduced in multiple experiments as indicated in the figure legends and in the 'statistics and reproducibility section in the method.
Randomization	For mouse studies: Mice were allocated randomly for the experiment.<br>For Human studies: nclusion/exclusion criteria are stated in method section and the human subjects (with experimental criterias) were recruited randomly for the experiment.
Blinding	Investigators in this study were not blinded for some of the in vivo and in vitro experiments since the effect of UVB on CPD dimers, melanin induction is well established and the phenotype is predetermined. Investigators were blinded for all ghrelin related experiments and quantifications.

Reporting for specific materials, systems and methods

We require information from authors about some types of materials, experimental systems and methods used in many studies. Here, indicate whether each material, system or method listed is relevant to your study. If you are not sure if a list item applies to your research, read the appropriate section before selecting a response.

Materials & experimental systems

n/a	Involved in the study
☐	☒ Antibodies
☐	☒ Eukaryotic cell lines
☒	☐ Palaeontology and archaeology
☐	☒ Animals and other organisms
☐	☒ Human research participants
☒	☐ Clinical data
☒	☐ Dual use research of concern

Methods

n/a	Involved in the study
☒	☐ ChIP-seq
☒	☐ Flow cytometry
☒	☐ MRI-based neuroimaging

Antibodies

Antibodies used

For western blot anti-ghrelin (Bioss, Cat# bs-1375R, 1:1000), anti-ER-α (Cell Signaling Technology, Cat# 8644, Clone D8H8, 1:1000), anti-NCOR1 (Cell Signaling Technology, Cat# 34271, Clone E4S4N), anti-p53 (Abcam, Cat# ab26, Clone PAb 240, 1:1000), and anti-β-actin (Cell Signaling Technology, Cat# 8457, Clone D6A8, 1:1000) primary antibodies were used. HRP-conjugated secondary antibodies: rabbit anti-mouse (Abcam, Cat# ab6820, 1:2000) or goat anti-rabbit (Abcam, Cat# ab97051, 1:2000 or Sigma-Aldrich, Cat# AP132P, 1:5000) were used.
For immunofluorescence section were stained using anti-ghrelin (Bioss, Cat# bs-1375R, 1:100), anti-Plin1 (Abcam, Cat# ab61682, 1:100), anti-CPD (Cosmo, Cat# CAC-NM-DND-001, Clone TDM2, 1:1000), anti-p53 (Cell Signaling Technology, Cat# 9282, 1:50) primary antibodies were used. Secondary antibodies used were Alexa Fluor 488 (Invitrogen, Cat#A11055, 1:1000), Alexa Fluor 594 (Invitrogen, Cat#A21203, 1:000), or Alexa Fluor 647 (Invitrogen, Cat#A31571, 1:000).
For ChiP experiments anti-p53 rabbit polyclonal antibody (Cell Signaling Technology, Cat# 9282, 1:100), anti-NCOR1 (Cell Signaling Technology, Cat# 34271, Clone E4S4N, 1:50), and normal rabbit IgG as control (Abcam, Cat# ab171870, 1:100) antibodies were used.

Validation

For IHC-P and western blot staining:
Ghrelin antibody (Bioss, Cat# bs-1375R) was validated using the ghrelin-positive MKN45 cells (data not shown). For the experimental application of this antibody, it was previously used by (Gao et al., 2016). As the prime source of the ghrelin, stomach tissues were also stained in the experiments to shown the validity of the antibody. ER-α antibody (Cell Signaling Technology, Cat# 8644) was validated by the manufacturer. Anti-CPD (Cosmo, Cat# CAC-NM-DND-001) antibody was validated in our study in presence of UVB radiation which is classically known to induce CPD damage (our previous study in Malcov et al., 2018). Anti-Plin1 (Abcam, Cat# ab61682) was validated (in our previous study Golan et al., 2019). All the HRP- conjugated secondary antibodies: rabbit anti-mouse (Abcam, Cat# ab6820) or goat anti-rabbit (Abcam, Cat# ab97051, or Sigma-Aldrich, Cat# AP132P) were validated by the manufacturer. All the secondary antibodies Alexa Fluor 488 (Invitrogen, Cat#A11055), Alexa Fluor 594 (Invitrogen, Cat#A21203), or Alexa Fluor 647 (Invitrogen, Cat#A31571) were validated by the manufacturer.

For ChiP experiments:
The p53 antibody for the ChiP grade was validated using the occupancy of the robust downstream target p21 in our experiments, NCOR1 antibody (Cell Signaling Technology, Cat# 34271) was already validated for ChiP experiments from the manufacturer.

Eukaryotic cell lines

Policy information about cell lines

Cell line source(s)

Primary human white subcutaneous pre-adipocytes (HWP; PromoCell) were from a female donor (Cat# C12730; Lot # 419Z023). 3T3-L1 (RRID:CVCL_0123) and HeLa cells (RRID:CVCL_0030) were obtained from ATCC. LiSa-2 cells were a gift from Peter Moeller (University of Ulm, Germany) under MTA, H1299 cells were obtained from ATCC and MKN45 cells were obtained from the lab of Professor Yossi Siloh (Tel Aviv University, Israel).

Authentication

The authentication of the pre-adipocytes (HWP, 3T3-L1 and LiSa-2) was done using the Oil Red O Staining for the validation of the lipid droplets. Other cell lines mentioned in the study were not authenticated by us.

Mycoplasma contamination

The cells were negative for the mycoplasma contamination.

Commonly misidentified lines
(See ICLAC register)

We have not used any misidentified cell lines in this study.

Animals and other organisms

Policy information about studies involving animals; ARRIVE guidelines recommended for reporting animal research

Laboratory animals

All mice used were from C57BL/6 background. All mice were housed in individually ventilated cages (IVC) (Maximum 5 mice per cage) for 12 hours dark/12 hours light phases with 22+1o C temperature and 32-35% humidity.
Wild-type C57BL/6 mice (males and females) aged 6-8 weeks were purchased from Envigo.
p53-knockout in mice
p53flx/flx mice were a gift from Eli Pikarksky (The Hebrew University of Jerusalem, Israel), and mice with the Fabp4 promoter directing expression of Cre recombinase (Fabp4Cre+) were purchased from Jackson Laboratory. These FABP4Cre+ transgenic mice were used as a Cre-lox tool for deletion of p53 floxed sequences in white adipose tissue. The p53 knockout in white adipose tissue was validated by genotyping.

	OVX mice<br>We performed the OVX and sham surgeries under the supervision of the Tel Aviv University Veterinarians and the OVX surgery was validated for the reduction of the circulating estrogen levels as mentioned in the manuscript.
Wild animals	No wild animals were used in the study.
Field-collected samples	No field-samples were collected in this study.
Ethics oversight	All animal experiments were performed in accordance with guidelines of the Tel Aviv University Institutional Animal Care and Use Committee with institutional policies and approved protocols.

Note that full information on the approval of the study protocol must also be provided in the manuscript.

Human research participants

Policy information about studies involving human research participants

Population characteristics	For Human cohort study<br>The human cohort study was approved by Tel Aviv University Ethics Committee under ethics number #0000668-2. Subjects were (aged 18-55 years) were recruited (self-volunteer) convenience sampling and were given the consent form with all the relevant information about the experiment. To avoid skin tone bias, all the participants in our study had Fitzpatrick Skin Type II-III. Since our study is to compare the solar UVB effects on males and females we took into consideration both genders. Subjects that were not included in the study were the pregnant women and metabolic diseases related – e.g. diabetes. No genotyping information was collected or tested during the experiment. The subjects on the past or current medications were noted by the medical doctor Dr. Tom Ben-Dov who performed the blood draws.<br><br>For UVB phototherapy questionnaire<br>UVB phototherapy questionnaire was conducted in the Tel Aviv Sourasky Medical Center and Assuta Hospital in Israel under approved Helsinki 0151-17-TLV and 17-ASMC-17. All the participants were recruited by convenience sampling and asked to sign an informed consent form. The sample consisted 43.7% males and 56.3% females. Data were collected through self-reported questionnaires (translated in Hebrew) before exposure to the UVB dose (T1) and10-12 exposure sessions for a month and after the treatment (T2). Genotyping-related information of the phototherapy subjects was neither collected nor tested in this study.
Recruitment	For Human cohort study<br>The human cohort study was approved by Tel Aviv University Ethics Committee under ethics number #0000668-2. Subjects were (aged 18-55 years) were recruited (self-volunteer) convenience sampling and were given the consent form with all the relevant information about the experiment. To avoid skin tone bias, all the participants in our study had Fitzpatrick Skin Type II-III.<br><br>For UVB phototherapy questionnaire<br>UVB phototherapy questionnaire was conducted in the Tel Aviv Sourasky Medical Center and Assuta Hospital in Israel under approved Helsinki 0151-17-TLV and 17-ASMC-17.Patients undergoing UVB phototherapy included phototherapy-responsive dermatoses including psoriasis, atopic dermatitis, mycosis fungoides, and general pruritus (aged 20–82). Skin tone directly affects the amount of UVB that penetrates the skin, and probably influences the response. To avoid this bias, most of the patients in our study had Fitzpatrick Skin Type II-III, and their treatment protocol was determined by the physician accordingly (i.e., higher skin tone will receive higher dose).
Ethics oversight	The human cohort study was approved by Tel Aviv University Ethics Committee under ethics number #0000668-2.<br>The UVB phototherapy questionnaire was conducted in the Tel Aviv Sourasky Medical Center and Assuta Hospital in Israel under approved Helsinki 0151-17-TLV and 17-ASMC-17.<br>The human skin explants from the patients undergoing abdominoplasty surgery at the Wolfson Medical Center, Israel were obtained under approved Helsinki number: 0015-16-WOMC.<br>All animal experiments were performed in accordance with the guidelines of the Tel Aviv University Institutional Animal Care and Use Committee with institutional policies and approved protocols (IACUC permit: 01-15-086 and 01-19-003).

Note that full information on the approval of the study protocol must also be provided in the manuscript.
